# The precision strategy of human genome correction via a set of circular donor DNA and its cleaver

**DOI:** 10.3389/fgeed.2026.1718252

**Published:** 2026-03-11

**Authors:** Kohji Kusano, Kaoru Takizawa, Jitsutaro Kawaguchi, Isamu Hara, Toyotaka Mori

**Affiliations:** 1 Tsukuba Factory, ID Pharma Co., Ltd., Tsukuba, Ibaraki, Japan; 2 ID Pharma Co., Ltd., Chiyoda-ku, Tokyo, Japan

**Keywords:** crossover-type homologous recombination, intra-cellular circular donor cleavage, natural replacement, safety distance, targeted duplication

## Abstract

Homologous recombination (HR) corrects a mutational sequence causing a genetic disease by replacing it with the normal sequence to restore a healthy state in humans. A targeted genomic breakage, such as that induced by CRISPR–Cas9, can trigger a copy-paste-type HR event; however, CRISPR–Cas9 more frequently induces imprecise non-homologous end-joining events, leading to one-step multiple knockout products for paralogous genes or homologous alleles, which can be considered a unique advantage. We have established a precision strategy for crossover-type HR-based gene editing, primed by intra-cellular circular donor cleavage (InCDC). The InCDC technique generates targeted duplication of the circular donor plasmid at the target locus in human cells, forming a doublet configuration comprising the donor DNA with the designed sequence and the target DNA with the original sequence, with much higher efficiency than conventional donor linearization techniques. This doublet form leads to the singlet form, resulting in retention of the designed allele. We found that the safety distance within the designed circular donor plasmid and its intra-cellular cleavage was particularly critical to protect a designed sequence from enzymatic exclusion, and we propose that InCDC technology enables precision genome editing, such as the replacement of a genetic disease-causing allele with the correctly designed allele.

## 1 Introduction

A DNA double-strand break (DSB) is defined as the concurrent cleavages of two close phosphodiester bonds of both strands of the double-strand DNA, inducing precise or imprecise non-homologous end-joining (NHEJ) (Roth et al., 1985; Rouet et al., 1994b; Hicks et al., 2010; Lin et al., 2013) and homologous recombination (HR)-directed DSB repair at its site on a chromosome (Resnick and Martin, 1976; Szostak et al., 1983; McGill et al., 1989; Rouet et al., 1994b; Nassif et al., 1994; Miura et al., 2012).

A targeted genomic breakage, such as ZFN (Morton et al., 2006), TALEN (Cermak et al., 2011), and CRISPR–Cas9 (Cong et al., 2013; Mali et al., 2013), which directs into a certain sequence in a locus of interest, seems to frequently produce small insertions and deletions (indels) at the targeted site as an outcome of imprecise NHEJ (Hicks et al., 2010). When a designed exogenous homologous DNA template is transfected, it is expected that a targeted genomic breakage is induced to paste a copy of the designed sequence of the homologous template into the target site through a synthesis-dependent strand annealing-type DSB repair mechanism (Nassif et al., 1994; Miura et al., 2012). However, there are several problems with targeted genomic breakage-related technology. (a) Off-target mutagenesis: The tools that employ this technology often break off-target sequences, especially when sequences similar to the target sequence exist in the genome, and produce potential off-target mutations (Fu et al., 2013; Frock et al., 2015), which may cause both undesired and unexpected side effects in patients receiving gene therapy. (b) On-target mutagenesis: Targeted genomic breakage induced precision repair at very low frequencies of approximately 2 × 10^−4^ per transfected cell (Miyaoka et al., 2014; Maruyama et al., 2015). It suggests that such techniques cause on-target imprecise NHEJ at a significantly higher frequency than on-target precise HR (copy-paste-type HR) with a designed exogenous DNA, making it difficult to obtain correct clones *ex vivo* for gene therapy. In the case of targeted genomic breakage-mediated precise genome editing from an *a*/*a* mutant homozygote to an *A* (normal allele)/*a* heterozygote at a putative disease-causing autosomal locus *A*, the first breakage hits the *a* allele to cause *indel*/*a* or *a*/*indel*, and then, the second breakage hits the other *a* allele to cause *indel*/*indel*, otherwise producing *indel*/*A* or *A*/*indel* (Kawamata et al., 2023), which may cause more severe symptoms than *a*/*a* in patients receiving gene therapy. For the above reasons, breakage-induced precision genome editing requires laborious PCR screening or several rounds of *sib* PCR screening to isolate designed clones. (c) Loss of heterozygosity (LOH) effects: In alleles on autosomal loci (2n), heterozygosity is maintained to preserve a healthy status in humans. When the targeted breakage is introduced into a locus during the G2 period (4n), its distal heterozygous statuses are possible to be changed to homozygous statuses in one of the daughter cells by the breakage-induced crossover-type HR with the counter locus (Miura et al., 2013), which may promote tumorigenesis due to LOH of tumor suppressor genes in patients receiving gene therapy. Such properties of CRISPR–Cas9 are particularly useful for one-step multiple gene knockouts of paralogous genes or homologous alleles in both basic and clinical research, representing a unique advantage—for example, in the generation of hypoimmunogenic cells with multiple knockouts of human leukocyte antigen genes (Kitano et al., 2022).

Conventional ends-in gene-targeting methods using a linearized donor plasmid (Hasty et al., 1991) were previously reported as a procedure to introduce a desired sequence or mutation into the target locus in murine embryonic stem (mES) cells (Wu et al., 1994). These methods circumvent the above host genome toxicities; however, critical observations related to the methods have been reported as follows: ends-out gene targeting in mES cells using linearized homologous DNA induced bidirectional extension of the homologous DNA, followed by its non-targeted integration (Mangerich et al., 2009). This report suggests caution against apparent targeted integration events in which DSB ends being prolonged by a break-induced replication mechanism (Anand et al., 2013; Wu and Malkova, 2021) are followed by non-targeted integration into chromosomes in mES cells. It remains unclear whether such apparent targeted integration (post-replication non-targeted integration) events occur in human cells, as well as in mES cells.

To address these issues, we focused on the Holliday junction-mediated HR pathway and have developed the intracellular circular donor cleavage (referred to as InCDC in this study)-induced precision genome editing method. This technology uses an exogenous circular donor DNA carrying a circular donor cleaver-recognizing sequence near the designed sequence and a gene expression system for the circular donor cleaver, which circumvents both on-target mutagenesis and LOH effects caused by targeted genomic breakage-related techniques. As a circular donor cleaver, we used the I-SceI meganuclease (Colleaux et al., 1988), the recognition sequence of which is not observed in databases of the human genome, thereby circumventing off-target mutagenesis and host genome toxicity (Rouet et al., 1994a; Soutoglou et al., 2007; Lin et al., 2015). The InCDC technology mediates targeted duplication reactions between the circular donor plasmid and its homologous locus, followed by natural replacement reactions of one allele with the other allele within the doublet configuration. Strong expression of the circular donor cleaver gene *nls*-*I-SceI* facilitates genuine targeted duplication events. During these processes, a single genetic element, the positive–negative selection fusion gene (Lupton et al., 1991; Schwartz et al., 1991), is useful for positively selecting targeted duplication clones and for negatively selecting natural replacement clones. In this study, the hypoxanthine phosphoribosyltransferase 1 (*HPRT*) locus, deficiency of which causes Lesch–Nyhan disease, neurobehavioral disorders, and biochemical defects due to increased *de novo* purine synthesis (Lesch and Nyhan, 1964; Seegmiller et al., 1967; Mastrangelo et al., 2012), was used to demonstrate the promotion of precision genome editing via InCDC technology.

## 2 Materials and methods

### 2.1 Tissue culture

The fibrosarcoma cell line HT1080 was used to evaluate circular donor DNA cleavage-induced targeted duplication and natural replacement. Other cell lines used in this study were derived from HT1080 cells. The cells were cultured in an atmosphere of 37 °C/5% CO_2_ in plastic tissue culture dishes and plates. Dulbecco’s modified Eagle’s medium (DMEM), supplemented with 10% fetal bovine serum and 1% penicillin/streptomycin, was used for routine passages and expansion of cells. Hygromycin (100 μg/mL) was used for the selection of donor DNA-integration clones, and 7.5 μg/mL 6-thioguanine was added for the selection of targeted duplication clones into the *HPRT* locus. Ganciclovir (1 μM; InvivoGen) or HAT supplement (50×) (Gibco) was used for the selection of natural replacement clones derived from a targeted duplication clone. For several experiments, advanced Dulbecco’s modified Eagle’s medium (advanced DMEM), supplemented with 1× GlutaMAX, 2% fetal bovine serum, and 1% penicillin/streptomycin, was used for routine passage and expansion of the cells. When an advanced DMEM-based culture medium was used, 50 μg/mL hygromycin, 7.5 μg/mL 6-thioguanine, and 0.1 μM ganciclovir were added for the selections. HT1080 and its derivatives were stored at −80 °C as follows: cultured cells were treated with a mixture of phosphate-buffered saline (PBS) and a dissociation reagent, 0.25 w/v% trypsin–1 mM EDTA (FUJIFILM Wako Pure Chemical Corporation), for 5 min at room temperature (trypsinization); the dissociation reaction was terminated by adding a 5- to 10-fold volume of DMEM-based culture medium, followed by pipetting and centrifugation, and the cells were resuspended in CELLBANKER 1 (TaKaRa Bio) solution, followed by storing several tubes (0.5–2 × 10^6^ cells/tube) at −80 °C.

### 2.2 Construction of circular donor plasmid vectors

pMB1KmHygTK: This plasmid is the vector to transfect a donor DNA. First, pSelect-zeo-HSV1tk (InvivoGen), cleaved by EcoRI and PstI to remove the *ZEO* gene, was ligated with the fragment including the Km^R^ gene constructed using KOD-PLUS-DNA polymerase (TOYOBO)-driving PCR on the pCR-BluntII-TOPO plasmid DNA (Invitrogen) with the primer pair 5′-CTT​AAT​TAA​CCT​GCA​GCC​GGA​ATT​GCC​AGC​TG-3′ and 5′-ATG​TGG​TAT​GGA​ATT​CGG​TGG​CCC​TCC​TCA​CGT​GC-3′ using the In-Fusion kit (TOYOBO) to obtain pSelect-Km-HSV1tk (1). On the other hand, pSelect-zeo-HSV1tk plasmid DNA (InvivoGen), cleaved by NcoI and SphI followed by blunt-ended treatment with T4 DNA polymerase with dNTPs at 11 °C, was ligated with the fragment including the *Hyg* gene constructed using KOD-PLUS-DNA polymerase (TOYOBO)-driving PCR on pcDNA3.1/Hygro plasmid with the primer pair 5′-TCA​CCG​GTC​ACC​ATG​AAA​AAG​CCT​GAA​CTC​ACC​GCG-3′ and 5′-TCA​AAG​GCA​GAA​GCA​ACT​TCT​ACA​CAG​CCA​TCG​GTC​C-3′ using the In-Fusion kit (TOYOBO) to obtain pSelect-zeo-HygTK (28–10), which contains the fusion gene of *Hyg* and *HSV1tk* genes. Finally, pSelect-Km-HSV1tk(1), cleaved by NotI and NheI to remove the *HSV1tk* gene, was ligated with the fragment including the *HygTK* gene constructed using KOD-PLUS-DNA polymerase (TOYOBO)-driving PCR on pSelect-zeo-HygTK(28-10) with the primer pair 5′-ATT​TAA​ATC​AGC​GGC​CGC​GGA​TCT​GCG​ATC​GCT​CCG-3′ (GT84) and 5′- TGT​CTG​GCC​AGC​TAG​CTC​AGG​TTT​AGT​TGG​CC-3′ using the In-Fusion kit (TOYOBO) to obtain pMB1KmHygTK (1).

pBS-HPRTEx2: This plasmid contains 5.4-kb of DNA covering the region containing intron 1, exon 2, intron 2, exon 3, and intron 3 of the *HPRT* locus. First, the 5′-2.8-kb fragment was constructed using KOD-PLUS-DNA polymerase (TOYOBO)-driving PCR on genomic DNA from the fibrosarcoma-derived cell line HT1080 with the primer pair 5′-AGC​CTG​GGC​AAC​ATA​GCG​AGA​CTT​C-3′ and 5′-TCT​GGT​CCC​TAC​AGA​GTC​CCA​CTA​TAC​C-3′, and the 3′-3.1-kb fragment was constructed using KOD-PLUS-DNA polymerase (TOYOBO)-driving PCR on genomic DNA from the fibrosarcoma-derived cell line HT1080 with the primer pair 5′-GCT​GGG​ATT​ACA​CGT​GTG​AAC​CAA​CC-3′ and 5′-TGG​CTG​CCC​AAT​CAC​CTA​CAG​GAT​TG-3’. There is a shared 0.5-kb sequence in the terminal end and the primed end of the 5′-2.8-kb fragment and the 3′-3.1-kb fragment, respectively. Finally, pBluescript SK+ (pBS) cleaved by NotI was ligated with the 5.4-kb fragment constructed using KOD-PLUS-DNA polymerase (TOYOBO)-driving PCR on both the 5′-2.8-kb fragment and the 3′-3.1-kb fragment with the primer pair 5′- TAG​TTC​TAG​AGC​GGC​CGC​AGC​CTG​GGC​AAC​ATA​GCG​AGA​CTT​C-3′ and 5′-CAC​CGC​GGT​GGC​GGC​CGC​TGG​CTG​CCC​AAT​CAC​CTA​CAG​GAT​TG-3′ using the In-Fusion kit (TOYOBO) to obtain pBS-HPRTEx2 (18-7). This plasmid was used as the template DNA for site-directed mutagenesis to introduce a designed sequence.

pBS-HPRTEx2ISCEI: This plasmid contains 5.4-kb DNA covering the region containing intron 1 and the *ISCEI* recognition sequence-included exon 2, intron 2, exon 3, and intron 3 of the *HPRT* locus. First, the 5′-2.5-kb fragment was constructed using KOD-PLUS-DNA polymerase (TOYOBO)-driving PCR on genomic DNA from the fibrosarcoma-derived cell line HT1080 with the primer pair 5′-TAG​TTC​TAG​AGC​GGC​CGC​AGC​CTG​GGC​AAC​ATA​GCG​AGA​CTT​C-3′ and the *ISCEI* recognition sequence (underlined)-tagged 5′-ATT​ACC​CTG​TTA​TCC​CTA​ACC​TGG​TTC​ATC​ATC​ACT​AAT​CTG-3′, and the 3′-2.9-kb fragment was constructed using KOD-PLUS-DNA polymerase (TOYOBO)-driving PCR on genomic DNA from the fibrosarcoma-derived cell line HT1080 with the primer pair *ISCEI* recognition sequence (underlined)-tagged 5′-TAG​GGA​TAA​CAG​GGT​AAT​TAT​GAC​CTT​GAT​TTA​TTT​TGC​ATA​CC-3′ and 5′-CAC​CGC​GGT​GGC​GGC​CGC​TGG​CTG​CCC​AAT​CAC​CTA​CAG​GAT​TG-3’. There is a shared *ISCEI* recognition sequence in the terminal end and the primed end of the 5′-2.8-kb and 3′-3.1-kb fragments, respectively. Finally, pBluescript SK + cleaved by NotI was ligated with the 5′-2.5-kb fragment and the 3′-2.9-kb fragment using the In-Fusion kit (TOYOBO) to obtain pBS-HPRTEx2ISCEI(21-1). This plasmid was used as the original plasmid to replace the fragment containing a designed sequence made by site-directed mutagenesis.

The sub-plasmid pBS-HPRTEx2Syn: This plasmid contains 5.4-kb DNA covering the region containing intron 1 and the synonymous sequence-included exon 2, intron 2, exon 3, and intron 3 of the *HPRT* locus. First, site-directed mutagenesis was performed using the KOD-PLUS-Inverse PCR mutagenesis kit (TOYOBO) with inverse primer pairs containing the synonymous sequence (underlined), 5′-GGC​TAC​GAT​CTC​GAC​CTC​TTT​TGC​ATA​CCT​AAT​CAT​TAT​GC-3′ and 5′-TGG​TTC​ATC​ATC​ACT​AAT​CTG-3′, and the template DNA pBS-HPRTEx2(18-7) plasmid to obtain pBS-HPRTEx2Syn(Inv15). Finally, pBS-HPRTEx2ISCEI(21-1), cleaved by BglII and SphI, was ligated with the synonymous sequence-included fragment constructed by digesting pBS-HPRTEx2Syn(Inv15) with BglII and SphI, followed by gel extraction to obtain pBS-HPRTEx2Syn (Inv15-2).

The sub-plasmid pBS-HPRT(Ex2Syn(1195)ISCEI): This plasmid contains 5.4-kb DNA covering the region containing intron 1, the synonymous sequence-incorporated exon 2, and the *ISCEI* recognition sequence-incorporated intron 2, exon 3, and intron 3 of the *HPRT* locus. First, site-directed mutagenesis was performed using the KOD-PLUS-Inverse PCR mutagenesis kit (TOYOBO) with inverse primer pairs *ISCEI* recognition-sequence (underlined)-tagged 5′-TAG​GGA​TAA​CAG​GGT​AAT​ATT​TTG​TAG​AAA​CAG​GGT​TCG​C-3′ and 5′-AAA​AAT​ATT​AGC​TGG​GAG​TGG-3′ and the template DNA pBS-HPRTEx2Syn (Inv15-2) plasmid to obtain pBS-HPRT(Ex2Syn(1195)ISCEI) (1).

The designed donor pMB1KmHygTK-HPRT(Ex2Syn(1195)ISCEI): The donor vector pMB1KmHygTK, cleaved by NotI, was ligated with the fragment with the synonymous sequence and the *ISCEI* recognition sequence, constructed by digesting pBS-HPRT(Ex2Syn(1195)ISCEI) (1) with NotI to obtain pMB1KmHygTK-HPRT(Ex2Syn(1195)ISCEI) (5). It was verified whether the direction of the *HPRT* sequence is identical to that of the *HygTK* gene.

The sub-plasmid pBS-HPRT(ISCEI(1195)Ex2Syn): This plasmid contains 5.4-kb DNA covering the region containing the *ISCEI* recognition sequence-incorporated intron 1 and the synonymous sequence-incorporated exon 2, intron 2, exon 3, and intron 3 of the *HPRT* locus. First, site-directed mutagenesis was performed using the KOD-PLUS-Inverse PCR mutagenesis kit (TOYOBO) with inverse primer pairs containing *ISCEI* recognition-sequence (underlined)-tagged 5′-TAG​GGA​TAA​CAG​GGT​AAT​CAA​AGC​ACT​GGG​ATT​ACA​AGT​G-3′ and 5′-GGA​GGC​TGA​GAC​AGG​AGA​GTT​GC-3′ and the template DNA pBS-HPRTEx2Syn (Inv15-2) plasmid to obtain pBS-HPRT(ISCEI(1195)Ex2Syn) (3).

The designed donor plasmid pMB1KmHygTK-HPRT(ISCEI(1195)Ex2Syn): The donor vector pMB1KmHygTK, cleaved by NotI, was ligated with the fragment with the synonymous sequence and the *ISCEI* recognition sequence, constructed by digesting pBS-HPRT(ISCEI(1195)Ex2Syn) (3) with NotI to obtain pMB1KmHygTK-HPRT(ISCEI(1195)Ex2Syn) (1). It is verified whether the direction of the *HPRT* sequence was identical to that of the *HygTK* fusion gene.

The sub-plasmid pBS-HPRT(ISCEI(545)Ex2Syn): This plasmid contains 5.4-kb DNA covering the region containing the *ISCEI* recognition sequence-incorporated intron 1 and the synonymous sequence-incorporated exon 2, intron 2, exon 3, and intron 3 of the *HPRT* locus. First, site-directed mutagenesis was performed using the KOD-PLUS-Inverse PCR mutagenesis kit (TOYOBO) with inverse primer pairs containing the *ISCEI* recognition-sequence (underlined)-tagged 5′-TAG​GGA​TAA​CAG​GGT​AAT​CAA​AGC​ACT​GGG​ATT​ACA​AGT​G-3′ and 5′-GGA​GGC​CGA​GGC​GGG​TGG​ATC​A-3′ and the template DNA pBS-HPRTEx2Syn (Inv15-2) plasmid to obtain pBS-HPRT(ISCEI(545)Ex2Syn) (4).

The designed donor plasmid pMB1KmHygTK-HPRT(Ex2Syn(545)ISCEI): The donor vector pMB1KmHygTK, cleaved by NotI, was ligated with the fragment containing the synonymous sequence and the *ISCEI* recognition sequence, constructed by digesting pBS-HPRT(ISCEI(545)Ex2Syn) (4) with NotI to obtain pMB1KmHygTK-HPRT(ISCEI(545)Ex2Syn) (1). It was verified whether the direction of the *HPRT* sequence is identical to that of the *HygTK* fusion gene.

The sub-plasmid pBS-HPRT(ISCEI (318)Ex2Syn): This plasmid contains 5.4-kb DNA covering the region containing the *ISCEI* recognition sequence-incorporated intron 1 and the synonymous sequence-incorporated exon 2, intron 2, exon 3, and intron 3 of the *HPRT* locus. First, site-directed mutagenesis was performed using the KOD-PLUS-Inverse PCR mutagenesis kit (TOYOBO) with inverse primer pairs containing the *ISCEI* recognition-sequence (underlined)-tagged 5′-TAG​GGA​TAA​CAG​GGT​AAT​TGT​ATT​TTT​AGT​AGA​GAC​GGG-3′ and 5′-AAA​AAA​TTA​GCC​GGG​TGT​GG-3′ and the template DNA pBS-HPRTEx2Syn (Inv15-2) plasmid to obtain pBS-HPRT(ISCEI (318)Ex2Syn) (2).

The designed donor plasmid pMB1KmHygTK-HPRT(Ex2Syn (318)ISCEI): The donor vector pMB1KmHygTK, cleaved by NotI, was ligated with the fragment containing the synonymous sequence and the *ISCEI* recognition sequence, constructed by digesting pBS-HPRTISCEI (318)Ex2Syn (2) with NotI to obtain pMB1KmHygTK-HPRT(ISCEI(318)Ex2Syn) (1). It was verified whether the direction of the *HPRT* sequence is identical to that of the *HygTK* fusion gene.

### 2.3 Construction of nls-I-SceI enzyme gene expression vectors

pCI-neo-nls-I-SceI: First, the I-SceI meganuclease gene was cloned from *Saccharomyces cerevisiae* W303 strain using KOD-PLUS-DNA polymerase (TOYOBO)-driving PCR on its genomic DNA with the primer pair nuclear localization signal sequence (underlined)-included 5′-GGA​TCC​TGC​AAA​GAT​GGA​TAA​AGC​GGA​ATT​AAT​TCC​CGA​GCC​TCC​AAA​AAA​GAA​GAG​AAA​GGT​CGA​ATT​GGG​TAC​CAT​GAA​AAA​TAT​TAA​AAA​AAA​TCA​AGT​AAT​GAA​TCT​GGG​TCC-3′ and *I-SceI* gene’s termination codon (underlined)-included 5′-ATG​CAT​TTA​TTT​TAA​AAA​AGT​TTC​GGA​TGA​AAT​AGT​ATT​AGG​C-3′. Next, the pCI-neo plasmid (Promega), cleaved by NotI, was ligated with the fragment including the *nls-I-SceI* gene constructed using the KOD-PLUS-DNA polymerase (TOYOBO)-driving PCR on its DNA with the primer 5′-GTC​GAC​CCG​GGC​GGC​CGC​CAT​GGA​TAA​AGC​GGA​ATT​AAT​TCC​CG-3′ and *I-SceI* gene’s termination codon (underlined)-included 5′-CTA​AAG​GGA​AGC​GGC​CGC​TTA​TTT​TAA​AAA​AGT​TTC​GG-3′ using the In-Fusion kit (TOYOBO) to obtain pCI-neo-*nls-I-SceI* (29-2) (DDBJ accession number to nls-I-SceI: LC920516).

nls-I-SceI-expression Sendai virus (SeV) vector: The RNA-dependent RNA polymerase of the SeV vector tends to make errors in the incorporation of ribonucleotide at A-rich sequences. To avoid this, primers for PCR were designed to target an A-rich site with a synonymous conversion from A/T to G/C. To construct the SeV-compatible I-SceI gene, the first PCR product was constructed using KOD-PLUS-Ver. 2-DNA polymerase (TOYOBO)-driving PCR on pCI-neo-nls-I-SceI (29-2) DNA with the primer pair 5′-CGA​GCC​TCC​AAA​gAA​GAA​GAG​AAA​GGT​CGA​ATT​GGG​TAC​CAT​GAA​AAA​TAT​TAA​gAA​gAA​TCA​AGT​AAT-3′ (NLS-I-SceIN_A36G_A78G_A81G_N) and 5′-GTT​CGG​GAT​GGT​TTT​cTT​GTT​GTT​AAC​G-3′ (NLS-I-SceI_A426G_C), and the second PCR product was constructed using KOD-PLUS-Ver. 2-DNA polymerase (TOYOBO)-driving PCR on pCI-neo-nls-I-SceI (29-2) DNA with the primer pair 5′-CGT​TAA​CAA​CAA​gAA​AAC​CAT​CCC​GAA​C-3′ (NLS-I-SceIN_A426G_N) and 5′-ATA​TGC​GGC​CGC​GAT​GAA​CTT​TCA​CCC​TAA​GTT​TTT​CTT​ACT​ACG​GTT​ATT​TTA​AAA​AAG​TTT​CGG​ATG-3′ (NLS-I-SceI_EIS_Not1_C). On these PCR products, the last PCR product was constructed using the same enzyme-driving PCR with the primer pairs NotI site-included 5′-ATA​TGC​GGC​CGC​GAC​GCC​ACC​ATG​GAT​AAA​GCG​GAA​TTA​ATT​CC*CGA​GCC​TCC​AAA​GAA​GAA​GAG​AAA​GGT​CG*-3′ (Not1_NLS-I-SceIN_A36G_N) and the same PCR as follows (NLS-I-SceI_EIS_Not1_C) to obtain the full length of nls-I-SceI DNA. Finally, a SeV vector plasmid DNA pSeV18+/TS15ΔF (Ban et al., 2011) cleaved by NotI, followed by BAP treatment, was ligated with the full DNA of nls-I-SceI cleaved by NotI to obtain pSeV18+*nls-I-SceI*/TS15ΔF. The SeV vector SeV18+nls-I-SceI/TS15ΔF was produced by reconstituting with this plasmid DNA as a template for RNA polymerization, followed by amplification.

### 2.4 Transfection

Preparation of exponentially growing cells: A cell stock (1 × 10^6^ cells) of HT1080 cells was seeded in a T75 flask with DMEM-based culture medium. Two or three days after seeding, the second culture was succeeded by the passage procedure as follows: the HT1080 cells were trypsinized, the dissociation reaction was terminated by adding a 10-fold volume of DMEM-based culture medium, followed by pipetting and centrifugation, and the cells were resuspended with a fresh medium. For the second culture, the recovered cells were plated at a cell density of 0.1–1.0 × 10^4^ cells/cm^2^ in T75 or T225 flasks with a fresh medium. When cells reached a cell density of 1.0–10 × 10^4^ cells/cm^2^ (2–3 days after plating), the exponentially growing cells were used for transfection by electroporation.

Electroporation: The exponentially growing HT1080 cells were trypsinized, the dissociation reaction was terminated by adding a 10-fold volume of DMEM-based culture medium, followed by pipetting and centrifugation, and the cells were resuspended in Opti-MEM for washing and finally resuspended in Opti-MEM at a concentration of 0.5–1 × 10^7^ cells/mL or 2 × 10^7^ cells/mL. An aliquot of 0.8 mL of the cell suspension was mixed with 10 μg of a designed circular donor plasmid DNA, which was purified with an adsorption column (QIAGEN) and dissolved in endotoxin-free TE, and 12.6 μg of an nls-I-SceI expression plasmid (pCI-neo-*nls-I-SceI* (29–2)) DNA or 13.3 μg of another nls-I-SceI expression plasmid (pCAG.I-SceI) DNA (Rouet et al., 1994a; Holkers et al., 2012), which was purified with an adsorption column (QIAGEN) and dissolved in endotoxin-free TE, or 1, 2, or 5 μg of the messenger RNA expressing nls-I-SceI enzyme, and was transferred into an electroporation cuvette (4 mm in width), followed by cooling on ice for 5 min. Otherwise, 10 μg of a linearized donor DNA alone was transferred into an electroporation cuvette (4 mm in width), followed by cooling on ice for 5 min. Each cuvette was subjected to three pulses of 140 volts/70 ms with 200 ms intervals using *Electro Square Porator BTX T820* (Artisan Technology Group), followed by cooling on ice for 5 min.

### 2.5 Cleaver expression tools for InCDC and donor DNA linearization

Plasmid-type nls-*I-SceI* gene expression vector: An intra-cellular cleaver expression plasmid, pCI-neo-*nls-I-SceI* (29-2), constructed in this study, or another nls-I-SceI expression plasmid, pCAG.I-SceI (Rouet et al., 1994a; Holkers et al., 2012) (bottom of lane v in Figure 2A; lane ii in Figure 3C), was used to cleave a designed circular donor DNA after transfection.

Messenger RNA expressing nls-I-SceI enzyme: This was prepared by T7 RNA polymerase-mediated *in vitro* transcription using the pCI-neo-*nls-I-SceI* (29-2) plasmid DNA as a template.

RNA virus-type nls-I-SceI enzyme expression vector: SeV18+*nls-I-SceI*/TS15ΔF was used to cleave a designed circular donor DNA in host cells. To prepare the freezing stocks of the HT1080 cells infected with the SeV vector, the second culture of HT1080 cells was spread onto T75 flasks at 4 × 10^6^ cells/T75 flask and incubated for approximately 24 h at 37 °C; then, the SeV vector was adsorbed with Opti-MEM onto the adherent cells at a multiplicity of infection (moi) of 3 at 32 °C [permissive temperature for replication of the TS15-type SeV vector (Ban et al., 2011)] by shaking the T75 flasks forth/back and left/right every 15 min for 2 hours. After viral adsorption, the SeV/Opti-MEM solution was removed, fresh Opti-MEM buffer was added to wash them, and then a fresh medium was added, followed by incubation at 32 °C/5% CO_2_ for 24 h to amplify the SeV vector genome and allow nls-I-SceI enzyme expression. HT1080/SeV cells were trypsinized, washed, and resuspended in Opti-MEM to allow recovery and finally resuspended in CELLBANKER 1 solution at a concentration of 2 × 10^6^ cells/mL; several tubes (2 × 10^6^ cells/tube) were then stored at −80 °C.

For transfection of a circular donor DNA, the stocks of the HT1080/SeV cells were plated and incubated at 37 °C (non-permissive temperature for replication of the TS15-type SeV vector) during the next different period (days): (1) plating onto four T225 flasks at 0.5 × 10^6^/T225, followed by incubation for approximately 96 h at 37 °C; (2) plating onto two T225 flasks at 1.0 × 10^6^/T225, followed by incubation for approximately 72 h at 37 °C; (3) plating onto two T225 flasks at 2.0 × 10^6^/T225, followed by incubation for approximately 48 h at 37 °C. Each of them reached 0.5–1.7 × 10^4^ cells/cm^2^ on T225 in cell density, and 4–13 × 10^6^ cells were recovered. These recovered cells were used to transfect a circular donor DNA by electroporation.

*ISCEI* site-linearized donor DNA: Each of the three types of *ISCEI* recognition site-containing designed donor plasmid DNA was digested with 1 unit of the I-SceI enzyme (NEB) per μg of DNA for 2 h at 37 °C and purified using an adsorption column (QIAGEN), followed by dissolution in endotoxin-free TE. The quality of *ISCEI*-linearized DNA was confirmed by agarose gel electrophoresis to verify linearization, and the quantity was confirmed compared with that of the circular donor DNA.

### 2.6 Isolation of donor-targeted duplication clones from hygromycin- and 6-thioguanine-resistant colonies

Selection of donor-targeted duplication clones: After transfection by electroporation with a circular donor plasmid DNA and an nls-I-SceI expression plasmid vector DNA, the cells were dispersed into 165 mL of pre-warmed DMEM and then plated at 5–10 × 10^6^ cells per 9-cm dish onto 16 dishes. Three days after electroporation, the medium was replaced with fresh medium containing hygromycin to select targeted duplication clones or random integration clones, which was repeated every 2 or 3 days. At 14–17 days after electroporation, hygromycin-resistant (Hyg^R^) colonies of two or four dishes out of 16 were counted to calculate the total number of Hyg^R^ colonies obtained, and then the medium was replaced with fresh medium containing hygromycin and 6-thioguanine to select targeted duplication clones showing an *HPRT*-deficient phenotype, which was repeated every 2 or 3 days. Twenty-four days after electroporation, hygromycin- and 6-thioguanine-resistant colonies from all dishes were counted. They were collected as HTG clones and allowed to grow in fresh medium containing the two drugs, followed by the preparation of cell-banker stocks and cell pellets for genomic DNA analysis, which were stored at −80 °C.

**FIGURE 1:**
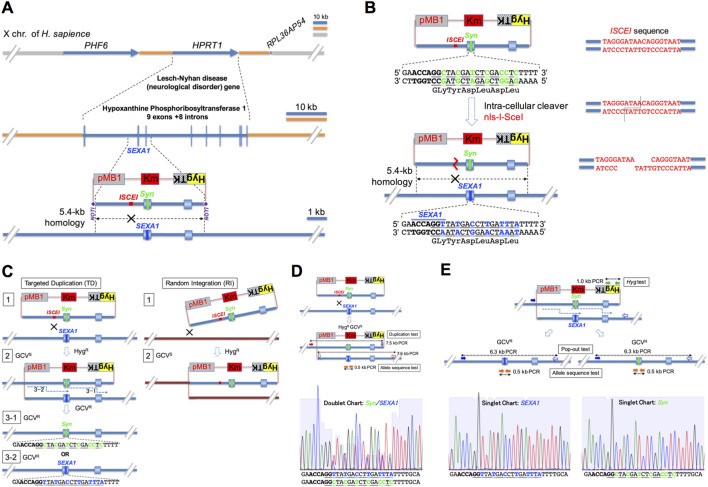
The assay system for human genome correction through intra-cellular circular donor cleavage-mediated targeted duplication and natural replacement. **(A)** The hypoxanthine phosphoribosyltransferase 1 (*HPRT1*) gene is located between the *PHF6* gene and the *RPL36AP54 microRNA* on the *X* chromosome in humans. It consists of nine exons and eight introns. The 5.4-kb section spanning intron 1, exon 2, intron 2, exon 3, and intron 3 was used as the targeting DNA, which is a probe used to search for homology across the entire chromosome. This 5.4-kb DNA is cloned into the pMB1KmHygTK plasmid vector (*Materials and Methods*), which has the bacterial replication origin (pMB1), the bacterial kanamycin-resistance gene (*Km*), and the fusion gene of the mammalian *Hyg* (hygromycin-resistant) gene with the *HSV1tk* (ganciclovir-sensitive) gene (*HygTK*). The I-SceI meganuclease-recognition site *ISCEI* and the designed sequence *Syn* are introduced into intron 1 and exon 2 of the cloned 5.4-kb DNA, respectively (*Materials and Methods*). *SEXA1*, labeled on exon 2 of the targeted locus, indicates the wild-type allele (*SEXA1* allele), which is replaced with the *Syn* sequence (*Syn* allele) in this assay system. The circular donor plasmid DNA consists of vector DNA (pink line) and homologous DNA (blue line). **(B)** A *Syn*-designed donor plasmid has synonymous codons (green letters) close to the *SEXA1* site and is transfected to human cells with an artificial intra-cellular cleaver gene *nls-I-SceI* expression vector. The *ISCEI* sequence (red letters) of the *Syn*-designed donor plasmid is cleaved by the nls-I-SceI endonuclease. **(C)** Targeted duplication (TD) pathway: Homologous recombination between a *Syn*-designed donor plasmid and its homologous region around the exon 2 (TD1) produces a targeted duplication clone (TD2), which has a duplication structure with the *Syn* sequence and the *SEXA1* sequence (*Syn*/*SEXA1* allele). The targeted duplication structure is naturally resolved through DNA replication during the S phase to form a single structure containing either the *Syn* allele (bold in TD3-1) or the *SEXA1* allele (bold in TD3-2). Random integration (RI) pathway: Non-homologous recombination between the *Syn*-designed donor plasmid and a random site (RI1) produces a random integration clone (RI2). **(D)** The circular *Syn*-designed donor plasmid accesses the targeted counter chromosomal region (upper) to form the *Syn*-retaining targeted duplication structure, *Syn*/*SEXA1* (center), which is confirmed by a couple of PCR-based tests as follows: (1) the duplication test is the PCR with the target-5′-outside forward primer (closed red arrow) and the plasmid-3′-inside reverse primer (closed violet arrow) and the other PCR with the plasmid-5′-inside forward primer (open violet arrow) and the target-3′-outside reverse primer (open red arrow); (2) the allele sequence test is the PCR with the Ex2–5′-outside primer and the Ex2–3′-outside primer (closed orange arrows), followed by its sequencing to verify the doublet chart of *Syn* and *SEXA1* allele sequences (lower). **(E)** The natural replacement structure containing the *Syn* allele or the *SEXA1* allele (center) is formed from a typical-targeted duplication structure (upper) by the popping-out of the circular DNA, which is confirmed using the following three tests: (1) the pop-out test is the PCR using target-5′-outside forward primer 2 (closed blue arrow) and target-3′-outside reverse primer 2 (open blue arrow) to verify the size characteristic of a natural replacement structure (the original size); (2) the *Hyg* test is the PCR with the 5′-*Hyg* forward primer and the 3′-*Hyg* reverse primer (closed green arrows) to verify the absence of the *Hyg* gene because the circular DNA that has popped out is removed by degradation or segregation in natural replacement cells; (3) the allele sequence test is the PCR with the Ex2–5′-outside forward primer and the Ex2–3′-outside reverse primer (closed orange arrows), followed by its sequencing to determine the sequence of the allele of a replacement clone, as shown in either a singlet chart of the *Syn* or *SEXA1* allele sequence (lower).

Analysis for genomic structure of donor-targeted duplication: Genomic DNA was extracted from each of the cell pellets of HTG clones, and then two PCR tests (duplication test; Figure 1D) were performed using the KOD-FX-DNA polymerase (TOYOBO) with one primer pair, plasmid-5′-inside forward primer 5′-TTG​CAA​GCA​GCA​GAT​TAC​GC-3′ (GT112), target-3′-outside reverse primer 5′-GCC​ACT​GCA​CCC​AGC​CGT​ATG​T-3′ (GT69), and the other primer pair, target-5′-outside forward primer 5’-ACG​ACC​TCG​CCC​GGC​CTT​GTA​TT-3′ (GT68), plasmid-3′-inside reverse primer 5′-AGC​TTC​AGC​TGT​GTT​CTG​GC-3′ (GT124), on the genomic DNA to verify the structure of targeted duplication into exon 2 at the *HPRT* locus, which consists of duplication of the donor DNA with the designed sequence and the recipient DNA with the original sequence, separated by the plasmid backbone containing the positive and negative selection marker pair. These primer pairs do not work in unexpected HTG clones, which are neither double-positive nor single-positive in the duplication test. For HTG clones with donor-targeted duplication, sequence analysis was performed by PCR using the KOD-FX-DNA polymerase with the primer pair, Ex2-5′-outside forward primer 5′-GCT​GGG​ATT​ACA​CGT​GTG​AAC​CAA​CC-3′ (GT19), Ex2-3′-outside reverse primer 5′-TCT​GGT​CCC​TAC​AGA​GTC​CCA​CTA​TAC​C-3′ (GT22), followed by a sequencing reaction on the PCR products (allele sequence test; Figure 1D). Duplication of the donor DNA with the designed sequence and the recipient DNA with the original sequence shows an overlapping sequencing chart (doublet chart) of the designed sequence and the original sequence. If an HTG clone shows a singlet chart of the original sequence, it indicates that the designed sequence has been excluded and, therefore, is not used to isolate designed donor replacement clones.

### 2.7 PCR screening of TD-containing dishes or TD-containing wells for hygromycin-resistant cells

Dish screening: After transfection with a circular donor plasmid DNA and an *nls-I-SceI* gene expression plasmid vector, the cells were dispersed into 165 mL of pre-warmed DMEM and then plated at 5∼10 × 10^6^ cells per 9-cm dish onto 16 dishes. Three days after electroporation, the medium was replaced with the fresh medium containing 100 μg/mL hygromycin to select targeted duplication clones or random integration clones, which was repeated every 2 or 3 days. At 14–17 days after electroporation, hygromycin-resistant (Hyg^R^) colonies of two dishes out of 16 were counted to calculate the total number of Hyg^R^ colonies obtained. The mixed cells from each dish were collected, followed by the preparation of cell-banker stocks and cell pellets for genomic DNA analysis, which were stored at −80 °C. One PCR test (TD screening; Figure 5B) was performed using the KOD-FX-DNA polymerase with a primer pair, plasmid-5′-inside forward primer 5′-TTG​CAA​GCA​GCA​GAT​TAC​GC-3′ (GT112) and target-3′-outside reverse primer 2 5′-TGG​GGA​AGG​AGG​CTA​TAG​AAG​ACC-3′ (GT330) on the genomic DNA to identify TD-containing dishes. This primer pair does not work in random integration clones. The other PCR test (*Syn*-TD screening; Figure 5B) was performed using the KOD-FX-DNA polymerase with a primer pair, target-5′-outside forward primer 2 5′- GCA​CAA​ACC​TCA​AAT​TTC​TCA​GCA​CTG​G-3′ (GT336) and *Syn* sequence (underlined)-included reverse primer 5′- GCA​AAA​GAG​GTC​GAG​ATC​GTA​GCC-3′ (GT340) on the genomic DNA to determine which of the TD dishes retain the *Syn* allele.

Well screening: The identified *Syn*-TD dish stock was plated and cultured for a few days. The pre-cultured *Syn*-TD dish cells were resuspended in DMEM-based culture medium and seeded at 2 cells/well onto three 96-well plates containing an HT1080 feeder or plated at 2 cells/well onto three fresh 96-well plates without a feeder. The HT1080 feeder plates were prepared before plating *Syn*-retaining TD dish-derived cells to improve the low clonal capacity of HT1080-derivative cells as follows: HT1080 pre-cultured cells were plated onto three 96-well plates at 64 cells/well in DMEM-based culture medium, and after 1-day incubation, they were used as HT1080-feeder plates. Six days after plating *Syn*-TD dish-derived cells onto 96-well plates, the medium was exchanged for DMEM-based culture medium with 100 μg/mL hygromycin; the resultant growing wells under hygromycin selection were transferred to 24-well plates and propagated, a small portion of which was used for genomic DNA extraction and stored at −80 as several cell stocks. PCR analysis was performed using the primer pairs for TD screening and *Syn*-TD screening to identify TD-containing wells and determine which of the TD wells retain the *Syn* allele, respectively. Each of the identified *Syn*-TD well stocks was plated onto a well in 6-well plates and cultured for a few days, followed by the preparation of cell stocks and cell pellet samples for genomic DNA analysis, which were stored at −80 °C.

### 2.8 Isolation of designed donor replacement clones

Selection of naturally generated donor-replacement clones: HTG clones with donor-targeted duplication containing a designed sequence were re-grown in the presence of hygromycin and 6-thioguanine from cell-banker stocks, pre-cultured without any drug for a few days, and then plated at 1 × 10^4^ cells per 15-cm diameter dish onto six dishes with DMEM-based culture medium or at 500 cells per 9-cm diameter dish onto 8 or 16 dishes with advanced DMEM-based culture medium. On day 5 or 6, the medium was replaced with fresh medium containing 1 μM ganciclovir with DMEM-based culture medium or 0.1 μM ganciclovir with advanced DMEM-based culture medium to select donor replacement clones three times per week by at most day 35. Ganciclovir-resistant (GCV^R^) colonies were collected as GCV clones and allowed to grow in fresh medium with the drug, followed by the preparation of cell-banker stocks and cell pellet samples for genomic DNA analysis, which were stored at −80 °C. In the case of HAT selection, 1× HAT supplement was added instead of GCV.

Analysis for genomic structure of designed donor replacement: Genomic DNA was extracted from each of the cell pellets of GCV clones, and then, a PCR test (*Hyg* test; Figure 1E) was performed with the primer pair 5′-*Hyg* forward primer 5′-TCA​CCG​GTC​ACC​ATG​AAA​AAG​CCT​GAA​CTC​ACC​GCG-3′ (GT38) and 3′-*Hyg* reverse primer 5′-TCA​AAG​GCA​GAA​GCA​ACT​TCT​ACA​CAG​CCA​TCG​GTC​C-3′ (GT39) on the *Hyg* structural gene and the genomic DNA to verify whether the plasmid backbone with the Hyg^R^ marker gene is removed from the target duplication structure. For the GCV clones, another PCR test (pop-out test; Figure 1E) was performed with the primer pair, target-5′-outside forward primer 2 5′-GCA​CAA​ACC​TCA​AAT​TTC​TCA​GCA​CTG​G-3′ (GT336) and target-3′-outside reverse primer 2 5′-TGG​GGA​AGG​AGG​CTA​TAG​AAG​ACC-3′ (GT330), targeting the sites outside the counter region of the 5.4-kb donor DNA and the genomic DNA to verify whether the plasmid backbone, with either single unit of the doublet form, was popped out from the target duplication structure. For the GCV clones, sequence analysis was performed by PCR with the primer pair, Ex2-5′-outside forward primer 5′-GCT​GGG​ATT​ACA​CGT​GTG​AAC​CAA​CC-3′ (GT19) and Ex2-3′-outside reverse primer 5′-TCT​GGT​CCC​TAC​AGA​GTC​CCA​CTA​TAC​C-3′ (GT22), across exon 2 of the *HPRT* gene, followed by a sequencing reaction on the PCR products (allele sequence test; Figure 1E), to determine whether it is the designed sequence or the target sequence. The duplication test was also performed to determine whether any duplicative structure remained within each of the GCV clones. The TK-deficient TD clones found as the GCV-resistant clones that are still doubly positive in the duplication test were analyzed by the PCR with the primer pair, 5′-ATT​TAA​ATC​AGC​GGC​CGC​GGA​TCT​GCG​ATC​GCT​CCG-3’ (GT84) and 5′-GCC​ACC​GAA​TTC​CAT​ACC​AC-3′, amplifying the region including the overall codes of the *HygTK* fusion gene, followed by sequencing, to identify *TK* mutations. When a GCV^R^ or HAT^R^ cell stock showed that the pop-out test was positive, but the *Hyg* test was weakly positive, or the allele sequence test was the doublet type of the *Syn* sequence and the *SEXA1* sequence, cell cloning was performed by plating at 1 or 2 cells/well onto 96-well plates, followed by propagation. Approximately 10–20 or fewer clones were analyzed by PCR and sequencing and classified into *Syn-*, SEXA1-, and TK-deficient TD clones.

## 3 Results

### 3.1 The model system to evaluate frequency and accuracy of human genome correction by InCDC-induced targeted duplication and natural replacement

The new concept of this precision strategy for human genome correction is to introduce a DSB within a circular donor plasmid to prime homologous recombination between the donor DNA and the target locus, whereas targeted genomic breakage, such as ZFN, TALEN, or CRISPR–Cas9, introduces a DSB at the target locus. This is the best solution to circumvent on-target mutagenesis due to DSB-induced non-homologous end-joining at the target locus, which is an issue of targeted genomic breakage-mediated genome editing. As the cleaver of the circular donor plasmid, we selected the I-SceI endonuclease (Colleaux et al., 1988), which recognizes a specific 18-bp sequence. This sequence is not in the databases of the human genome. The high specificity of this enzyme for the recognition sequence has been demonstrated as follows: human cells transfected with I-SceI showed background levels of DSB signals (Lin et al., 2015); Mammalian cells showed no signals of I-SceI-induced DSB in the genome (Soutoglou et al., 2007); cellular toxicity with I-SceI expression was not observed in mammalian cells (Rouet et al., 1994a). Thus, this enzyme is a suitable tool to circumvent off-target mutagenesis, which is another issue associated with targeted genomic breakage-mediated genome editing.

To construct a model system to evaluate the frequency and accuracy of such InCDC-mediated genome editing, we used the HT1080 cell line derived from a male individual as human cells and constructed the model system comprising a target site for human genome correction, the region around exons 2 and 3 at the *HPRT* locus (middle in Figure 1A) in the X chromosome (upper in Figure 1A), and a designed donor DNA, which covers the 5.4-kb section across introns 1–3 (lower in Figure 1A).

**FIGURE 2:**
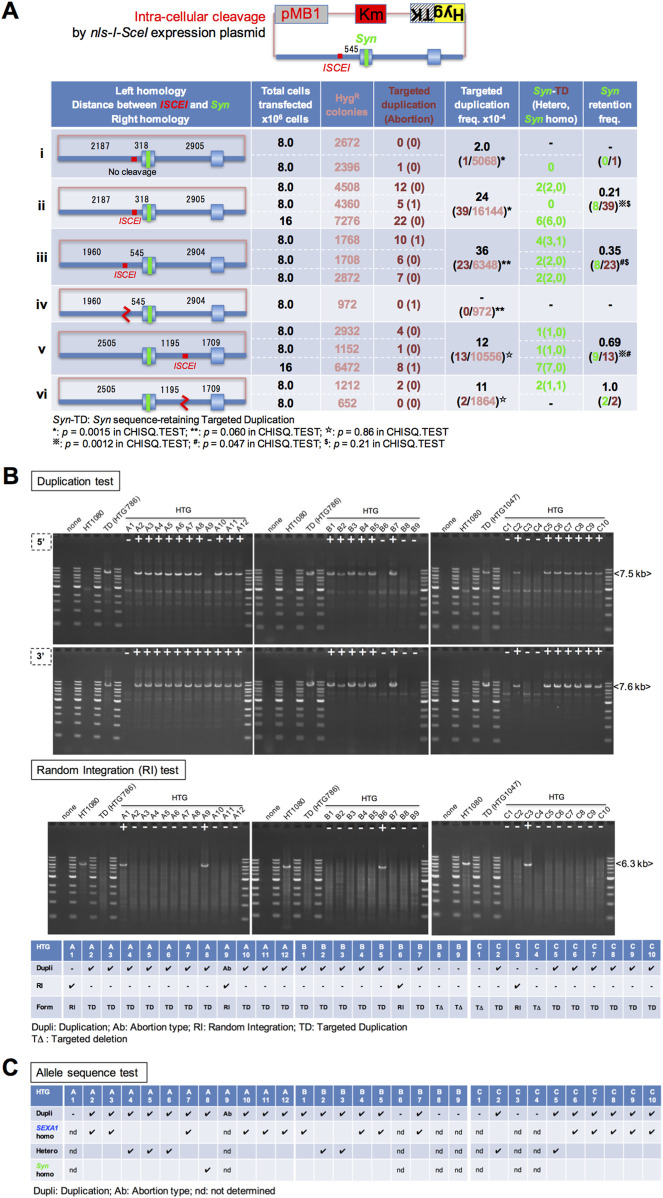
Several hundred base pairs in distance between the cleavage site and the designed sequence on a circular donor plasmid are required to retain the designed allele. **(A)** A circular donor plasmid, pMB1KmHygTK-HPRT(ISCEI(318)Ex2Syn), pMB1KmHygTK-HPRT(ISCEI(545)Ex2Syn), or pMB1KmHygTK-HPRT(Ex2Syn(1195)ISCEI), consists of the vector DNA (pink line) and the homologous DNA (blue line) with the *Syn* sequence and the *ISCEI* cleavage site. In the left column, three types of circular donor plasmids depict the length of left homology, the distance between the cleavage point and the *Syn* sequence, and the length of right homology with a cleavage type (no cleavage, intra-cellular cleavage, or linearized at the *ISCEI* site). In the following three columns, the numbers show the total cells transfected by electroporation, the whole integration clones observed as hygromycin-resistant (Hyg^R^) colonies, the TD clones, and the abortion clones in brackets, which were doubly positive and singly positive in the duplication test (Figure 1D), respectively. In the last three columns, the frequency of the TD clones relative to the whole Hyg^R^ colonies, the number of the *Syn*-retaining TD clones, and the frequency of the *Syn*-retaining TD clones relative to the TD clones are shown. In the case of the intra-cellular cleavage type, the nls-I-SceI expression plasmid, pCI-*nls-I-SceI* or pCAG.I-SceI plasmid (bottom on lane v), was co-transfected with a circular donor DNA (*Materials and Methods*). The calculations by CHISQ.TEST (*Excel*) are shown in Supplementary Table 1. **(B)** The duplication test for the clones derived from the donor plasmid with HPRT(ISCEI(545)Ex2Syn): the results from the duplication test (Figure 1D) for 12 hygromycin-resistant and 6-thioguanine-resistant (Hyg^R^TG^R^) clones (HTG-A1–A12) out of 1,768 Hyg^R^ clones (top of lane iii in panel **(A)**) are shown. Out of 12, 10 clones are doubly positive, 1 clone (HTG-A9) is singly positive (Ab: Abortion), and 1 clone (HTG-A1) is doubly negative in the duplication test. The results for 9 Hyg^R^TG^R^ clones (HTG-B1–B9) out of 1708 Hyg^R^ clones [Center of lane iii in **(A)**] are shown. Out of 9, 6 clones are doubly positive and 3 clones (HTG-B5, B8, and B9) are doubly negative in the test. The results for 10 Hyg^R^TG^R^ clones (HTG-C1–C10) out of 2,872 Hyg^R^ clones (bottom of lane iii in panel A) are shown. Out of 10, 7 clones are doubly positive and 3 clones (HTG-C1, C3, and C4) are doubly negative in the test. Random integration (RI) test for the clones derived from the donor plasmid with HPRT(ISCEI(545)Ex2Syn): The results from the RI test for HTG-A1–A12, HTG-B1–B9, and HTG-C1–C10 clones are shown, based on the PCR assay with target-5′-outside forward primer 2 (closed blue arrow) and target-3′-outside reverse primer 2 (open blue arrow), which is the same primer pair as that in the pop-out test (Figure 1E). TD shows to be doubly positive in the duplication test and negative in the RI test. RI shows to be positive in the RI test and singly positive (HTG-A9) or doubly negative (HTG-A1, B6, and C3) in the duplication test. TD (targeted deletion) shows to be doubly negative in the duplication test and negative in the RI test (HTG-B8, B9, C1, and C4). HTG786 is a TD clone with 5′*SEXA1*/*Syn*3’. HTG1047 is a TD clone with 5′*Syn*/*SEXA1*3’. **(C)** The allele sequence test for the clones derived from the donor plasmid with HPRT(ISCEI (545)Ex2Syn): The results from the allele sequence test (Figure 1D) for the HTG clones, which are doubly positive in the duplication test, are summarized as *SEXA1* homo (5′*SEXA1*/*SEXA1*3′), hetero (5′*Syn*/*SEXA1*3′ or 5′*SEXA1*/*Syn*3′), and *Syn* homo (5′*Syn*/*Syn*3′). The results from the allele sequence test are summarized in Supplementary Table 2.

The designed sequence consists of six continuous synonymous codons (*Syn* allele sequence: upper in Figure 1B), in which the six original codons (*SEXA1* allele sequence: lower in Figure 1B) are replaced. The unique double-strand break site *ISCEI*, which is the I-SceI meganuclease recognition sequence (right in Figure 1B), is positioned at 318 bp, 545 bp, or 1195 bp from the designed sequence *Syn* in a designed donor DNA (Figure 2A). When nls-I-SceI enzymes, to which a nuclear localization signal was added (*Materials and Methods*), are expressed in a transfected cell, a circular donor plasmid is cleaved at its *ISCEI* site (right in Figure 1B).

It is well known that targeted duplication inserts the whole donor plasmid DNA into the target site through crossover-type homologous recombination (Hasty et al., 1991; Brenneman et al., 1996). As homology-directed DNA repair is known to occur in the late S and G2 phases of the cell cycle (Smirnikhina et al., 2022), the exponentially growing HT1080 cells were used for transfection with donor plasmid DNA (*Materials and Methods*). The cleavage of circular donor plasmid mediates the integration into the target region around exons 2–3 at the *HPRT* locus (blue-lined chromosome in TD1; Figure 1C) and forms the configuration of a duplication of the donor DNA with the designed sequence *Syn* and the target DNA with the original sequence *SEXA1*, intervened by the plasmid vector bearing the positive and negative selection fusion gene (*HygTK*) (targeted duplication product: TD2). The targeted duplication clones were isolated from the positively selected clones [hygromycin-resistant (Hyg^R^) colonies]. *HPRT*-defective clones exhibit resistance to a guanine analog 6-thioguanine. Taking advantage of this *HPRT* ^*-*^ phenotype, hygromycin/6-thioguanine-double-resistant (Hyg^R^Tg^R^) colonies, as candidates for *HPRT* exon 2-targeted duplication clones, were analyzed by PCR with a certain primer pair (Figure 1D). Finally, out of targeted duplication clones (TD2), natural replacement clones can be generated as the negatively selected clones (natural replacement product: TD3-1 or -2 in Figure 1C), which are verified by PCR and sequencing for the presence of the singlet allele *Syn* out of the negatively selected clones (GCV^R^ colonies). The natural replacement reaction is associated with popping-out of the circular plasmid DNA containing the *HygTK* gene and either the designed or the original allele. Thus, the natural replacement clones (TD3-1 or -2) do not contain the *HygTK* gene.

Several reports on random integration of foreign DNA indicate the involvement of microhomology-mediated conjugation (Yan et al., 2010; Yan et al., 2013). Circular or cleaved donor DNA integrates at sites on chromosomes (brown-lined chromosome: RI1 in Figure 1C), which is mostly generated through microhomology-mediated conjugation. A positively selected clone (Hyg^R^) has the resulting typical random integration structure (random integration product: RI2), comprising the plasmid DNA bearing the selection marker gene (*HygTK*) and the *Syn*-designed donor sequence but was never isolated by PCR using a certain primer pair for the targeted duplication structure (Figure 1D).

To verify a targeted duplication structure (TD2 in Figure 1C) of Hyg^R^Tg^R^ clones, the duplication test (Figure 1D) is performed by PCR with one primer pair, which is positioned at the 5′-outside genomic region of donor homologous DNA (a forward primer) and the 3′-inside plasmid region of donor homologous DNA (a reverse primer), and with the other primer pair, which is positioned at the 5′-inside plasmid region of donor homologous DNA (a forward primer) and the 3′-outside genomic region of donor homologous DNA (a reverse primer). Next, to verify whether a duplicative structure is hetero-allelic (*Syn* and *SEXA1*) (TD2), the allele sequence test (Figure 1D) is performed by PCR and sequencing. A typical result of this test shows a doublet chart, including double-peaks of the six synonymous and original codons, which are identical in height (lower in Figure 1D).

To verify a natural replacement structure (TD3-1 or -2) of GCV^R^ colonies, the pop-out test (Figure 1E) is performed by PCR using the PCR primer pair, which is positioned at the 5′-outside genomic region of donor homologous DNA (a forward primer) and the 3′-outside genomic region of donor homologous DNA (a reverse primer), followed by the allele sequence test to verify whether a natural replacement clone is *Syn* (TD3-1) or *SEXA1* (TD3-2) by PCR and sequencing. Concurrently, we verified the absence of plasmid DNA generated by a popping-out reaction during a natural replacement event, as indicated by the absence of PCR products using a primer pair targeting the *Hyg* structural gene sequence. If 1.0-kb PCR products on the *Hyg* gene are detected and 6.3-kb PCR products across the target region are not detected in a GCV^R^ colony, it is supposed to be TK-deficient derivatives from the targeted duplication clone (TD2).

### 3.2 Intra-cellular circular donor cleavage leads to targeted duplication, resulting in the formation of a doublet configuration comprising the designed sequence and the target sequence in human cells

Conventional ends-in gene targeting methods using linearized donor plasmid (Hasty et al., 1991) were reported as the procedure leading to the replacement of the target sequence with a desired sequence in mES cells (Wu et al., 1994). However, the report suggests caution that post-replication non-targeted integration (apparent targeted integration) events occur in mES cells (Mangerich et al., 2009). Regarding it as a critical issue, we examined whether in human cells, ends-in gene targeting-based methods produce such apparent targeted integration using the assay system (Figure 1).

When a circular donor plasmid pMB1KmHygTK-HPRT(ISCEI(545)Ex2Syn) was transfected with *nls-I-SceI* gene expression plasmid, the TD clones appear to be obtained at a frequency of 3.6 × 10^−3^ (23/6348 Hyg^R^) per overall-integration clone (lane iii in Figure 2A), based on the criteria to be doubly positive in the duplication test (Figure 1D) (upper of Figure 2B).

These candidates for TD clones were examined using the random integration (RI) test (lower in Figure 2B), which is a PCR assay with target-5′-outside forward primer 2 (closed blue arrow) and target-3′-outside reverse primer 2 (open blue arrow), being the same primer pair as in the pop-out test (Figure 1E). If a TD candidate clone is negative in the RI test, it is considered a targeted duplication clone. All of the 23 TD candidate clones were shown to be negative in the PCR assay (Figure 2B). These results indicate that all of these TD structures (doubly positive in the duplication test) are at the target site. The abortion-type (singly positive in the duplication test) clone (HTG-A9 in Figure 2B) was positive in the RI test. This clone appears to be the outcome of a post-replication non-targeted integration, as is the case of mES cells (Mangerich et al., 2009). We conclude that, in HT1080 cells, post-replication non-targeted integration events are rare, and their frequency is far lower than that of TD events.

These 23 TD clones were examined using the allele sequence test (Figure 1D) to identify the TD clones retaining the designed sequence *Syn*. Out of 23, 7 clones were associated with the hetero-duplication, which consisted of the designed sequence *Syn* and the target sequence *SEXA1*, and one clone was associated with the homo-duplication, which consisted of only the designed sequence *Syn* (Figure 2C). The *Syn* retention frequency per TD clone was 35% (8/23) for the circular donor cleavage at the *ISCEI* site, located 545 bp away from the *Syn* allele sequence (lane iii in Figure 2A).

It is later discussed how each of the four types of allelic duplications [15 clones out of 23 TD clones: 5′*SEXA1*/*SEXA1*3’ (Figure 7C); 5 clones: 5′*Syn*/*SEXA1*3’ (Figure 7D); 2 clones: 5′*SEXA1*/*Syn*3’ (Figure 7E); 1 clone: 5′*Syn*/*Syn*3’ (Figure 7F)] is generated in InCDC-mediated crossover-type HR. These four types were determined using 5'/3′*Syn* primer PCR analysis (Figures 7A,B).

When a circular donor plasmid pMB1KmHygTK-HPRT(ISCEI(318)Ex2Syn) was transfected without any expression tool of the nls-I-SceI enzyme, the targeted duplication clones were obtained at a frequency of 2.0 × 10^−4^ (1/5068 Hyg^R^) per overall-integration clone (lane i in Figure 2A). When the same circular donor plasmid pMB1KmHygTK-HPRT(ISCEI(318)Ex2Syn) was transfected with the plasmid expressing nls-I-SceI enzyme, the targeted duplication clones were obtained at a frequency of 2.4 × 10^−3^ (39/16184 Hyg^R^) per overall-integration clone, which significantly increased (12-fold) compared with that without expression of the *nls-I-SceI* gene (*: lane i vs. lane ii in Figure 2A). These results indicate that circular donor cleavage at the *ISCEI* site is crucial for priming targeted duplication events.

Out of 39 targeted duplication clones generated using the circular donor cleavage of pMB1KmHygTK-HPRT(ISCEI (318)Ex2Syn), 8 clones were associated with hetero-duplication, which consisted of the donor DNA with the designed sequence *Syn* and the target DNA with the original sequence *SEXA1* (5′*Syn*/*SEXA1*3′); however, the other 31 clones were associated with the homo-duplication (5′*SEXA1*/*SEXA1*3′) (Figure 2A). The *Syn* retention frequency per TD clone was 21% (8/39) for the case of the circular donor cleavage at the *ISCEI* site, located 318 bp away from *Syn* (lane ii in Figure 2A).

When the circular donor plasmid pMB1KmHygTK-HPRT(Ex2Syn(1195)ISCEI) was transfected with the plasmid expressing nls-I-SceI enzyme, the targeted duplication clones were obtained at a frequency of 1.2 × 10^−3^ (13/10556 Hyg^R^) per overall-integration clone (lane v in Figure 2A). Furthermore, 9 clones out of 13 clones were associated with the hetero-duplication, which consisted of the donor DNA with the designed sequence *Syn* and the original sequence *SEXA1*. The *Syn* retention frequency per TD clone was 69% (9/13) for the circular donor cleavage at the *ISCEI* site, located 1,195 bp away from *Syn* (lane v in Figure 2A).

Based on *Syn*-retention frequencies per TD clone (lanes ii, iii, and v in Figure 2A), *Syn* deletion frequencies per TD clone were 79% [(39-8)/39], 65% [(23–8)/23], and 31% [(13-9)/13] in each of the circular donor-plasmids with HPRT[ISCEI (318)Ex2Syn], HPRT[ISCEI (545)Ex2Syn], and HPRT[Ex2Syn (1195)ISCEI], respectively. The *Syn*-retention frequency per TD clone in the circular donor-plasmid with HPRT[Ex2Syn (1195)ISCEI] is significantly higher than that with HPRT[ISCEI (318)Ex2Syn] or HPRT[ISCEI (545)Ex2Syn] (^※^: lane ii vs. lane v; ^#^: lane iii vs. lane v in Figure 2A). These results imply that the circular donor plasmid is indeed cleaved at the *ISCEI* site to prime targeted duplication events, and a putative exonuclease attack occurs immediately after intra-cellular cleavage against the circular donor plasmid to cause the removal of the designed sequence. Thus, the concept of “Safety Distance”, in which the designed sequence is placed several hundred base pairs away from the *ISCEI* site in a circular donor plasmid to protect it from a putative exonuclease attack, is crucial to obtain designed sequence-retaining TD clones.

### 3.3 Efficiency in targeted duplication: intra-cellular circular donor cleavage vs. donor linearization

The donor plasmid linearized with the commercial I-SceI enzyme was electroporated, but this technique is less successful in obtaining the targeted duplication clones (lanes iv and vi in Figure 2A) compared with the InCDC technique (lanes iii and v in Figure 2A). We approach why linearized donor DNA is less successful in targeted duplication by quantifying the overall integration frequency per transfected viable cell. In this approach, an advanced DMEM-based culture medium was used to obtain a more corrected value of transfected viable cells as the clonal capacity of the advanced DMEM-based culture medium is greater than that of the DMEM-based culture medium (*Materials and Methods*).

**FIGURE 3:**
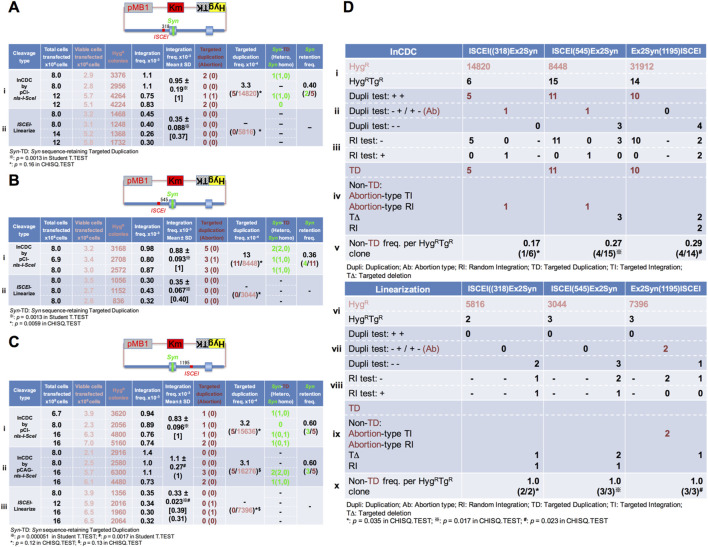
InCDC is required for efficient targeted duplication. **(A–C)** A circular donor plasmid, pMB1KmHygTK-HPRT(ISCEI(318)Ex2Syn) **(A)**, pMB1KmHygTK-HPRT(ISCEI (545)Ex2Syn) **(B)**, or pMB1KmHygTK-HPRT(Ex2Syn (1195)ISCEI) **(C)**, along with plasmid DNA expressing the intra-cellular cleavage enzyme nls-I-SceI, was electroporated into the total number of cells transfected indicated in the first column. Alternatively, donor DNA linearized at the *ISCEI* site alone was electroporated into the total number of cells indicated in the first column. In the next five columns, the numbers show the viable cells transfected, which were calculated by multiplying the number of total cells plated by CFU (colony forming units)/500 total cell on two 9-cm diameter dishes; the overall integration clones as Hyg^R^ colonies generated; the overall integration frequency as the fraction of the Hyg^R^ colonies generated relative to the viable cells transfected; and the targeted duplication clones and the abortion-type clones in brackets, which were doubly positive and singly positive in the duplication test for Hyg^R^Tg^R^ colonies, respectively. In the last three columns, the frequency of the targeted duplication clones per overall integration (Hyg^R^) clone, the numbers of the *Syn*-retaining targeted duplication clones, and the frequency of the *Syn*-retaining targeted duplication clones per targeted duplication clone are shown. In these experiments, an advanced DMEM-based culture medium was used to obtain a more corrected value of transfected viable cells as the clonal capacity of the advanced DMEM-based culture medium is greater than that of the DMEM-based culture medium (*Materials and Methods*). The calculations using the Student T-TEST (*Excel*) and CHISQ.TEST (*Excel*) in **(A–C)** are shown in Supplementary Table 3. The results from the allele sequence test in **(A–C)** are summarized in Supplementary Table 4. **(D)** In the “InCDC” table (upper) and “Linearization” table (lower), lanes i and vi show the number of Hyg^R^ clones and Hyg^R^Tg^R^ clones generated, respectively; lanes ii and vii show the number of Hyg^R^Tg^R^ clones classified into three types using the duplication test (Figure 2B); lanes iii and viii show each of the Hyg^R^Tg^R^ clones also classified into two types using the RI test (Figure 2C); lanes iv and ix show the number of each of the forms [TD or non-TD (abortion-type TI and abortion-type RI, TD, or RI] of Hyg^R^Tg^R^ clones classified, determined based on the two tests above; and lanes v and x show the non-TD frequency per overall integration (Hyg^R^) clone. The calculations using the CHISQ.TEST (*Excel*) in panel **(D)** are shown in Supplementary Table 3.

When a circular donor plasmid pMB1KmHygTK-HPRT(ISCEI(318)Ex2Syn) was transfected with the plasmid expressing nls-I-SceI enzyme, the overall integration (Hyg^R^) clones were obtained at a frequency of 0.95 × 10^−3^ per transfected viable cell (lane i in Figure 3A). Alternatively, when the linearized donor plasmid pMB1KmHygTK-HPRT(ISCEI(318)Ex2Syn) was transfected alone, the overall integration (Hyg^R^) clones were obtained at a frequency of 0.35 × 10^−3^ per transfected viable cell (lane ii in Figure 3A), which significantly decreased (0.37-fold) compared with the co-transfection of the circular donor plasmid and the plasmid DNA expressing nls-I-SceI enzyme (lanes i and ii in Figure 3A).

When another donor plasmid, pMB1KmHygTK-HPRT(ISCEI(545)Ex2Syn) or pMB1KmHygTK-HPRT(Ex2Syn(1195)ISCEI), was used, the donor linearization technique significantly decreased (0.31–0.40-fold) compared with that using the InCDC technique in overall integration frequency per transfected viable cell (lanes i and ii in Figure 3B; lanes i and ii in Figure 3C).

Taking together these overall integration frequencies, the linearized donor DNA appears to be more susceptible to digestion in a cell than the circular donor plasmid co-transfected with nls-I-SceI expression plasmid DNA.

When the circular donor plasmid pMB1KmHygTK-HPRT(ISCEI(318)Ex2Syn) was transfected, the targeted duplication clones were obtained at a frequency of 3.3 × 10^−4^ (5/14820 Hyg^R^: lane i in Figure 3A) per overall integration clone using the InCDC technique but were not obtained using the donor linearization technique (0/5816 Hyg^R^: lane ii in Figure 3A).

When the circular donor plasmid pMB1KmHygTK-HPRT(ISCEI(545)Ex2Syn) was transfected, the targeted duplication clones were obtained at a frequency of 1.3 × 10^−3^ (11/8448 Hyg^R^: lane i in Figure 3B) per overall integration clone using the InCDC technique but were not obtained using the donor linearization technique (0/3044 Hyg^R^: lane ii in Figure 3B), for which statistical analysis showed that the TD frequency per overall integration clone with InCDC significantly increased compared with that using the donor linearization technique (*: lane i vs. lane ii in Figure 3B).

When the circular donor plasmid pMB1KmHygTK-HPRT(Ex2Syn(1195)ISCEI) was transfected, the targeted duplication clones were obtained at the frequencies of 3.2 × 10^−4^ and 3.1 × 10^−4^ (5/15636 Hyg^R^; 5/16276 Hyg^R^: lanes i and ii in Figure 3C) per overall integration clone using the InCDC technique but were not obtained using the donor linearization technique (0/7396 Hyg^R^: lane iii in Figure 3C).

Taking together these TD frequencies, the InCDC technique is more efficient and successful in obtaining the targeted duplication clones than the donor linearization technique.

We confirmed that *Syn*-retaining TD clones were identified among the TD clones derived from each of the three types of circular donor plasmid using the InCDC techniques (2 of 5: lane i in Figure 3A; 4 of 11: lane i in Figure 3B; 3 of 5 and 3 of 5: lanes i and ii in Figure 3C).

We also analyzed using the RI test (Figure 2B) and the duplication test (Figure 1D) for the Hyg^R^Tg^R^ clones generated by each of the InCDC and donor linearization techniques to determine their final forms (targeted duplication, abortion-types, targeted deletion, and random integration), as summarized in Figure 3D. The forms except TD (non-TD forms) derived from InCDC were generated at the frequencies of 1/6, 4/15, and 4/14 per Hyg^R^Tg^R^ clone (lane v in Figure 3D); the non-TD forms derived from donor linearization were generated at the frequencies of 2/2, 3/3, and 3/3 per Hyg^R^Tg^R^ clone (lane x in Figure 3D). We statistically analyzed them to evaluate InCDC vs. linearization in gene editing accuracy and demonstrated that InCDC was more accurate than linearization (*: lane v vs. lane x; ^※^: lane v vs. lane x; ^#^: lane v vs. lane x in Figure 3D).

We found that the donor linearization technique resulted in lower overall integration frequencies and higher non-TD frequencies and was inefficient for generating targeted duplication. We also found that InCDC genome editing generated non-TD clones and TD clones, but the TD (HR) clones were more frequent than non-TD (non-HR) clones. This contrasts with the fact that CRISPR–Cas9 generated far more Indel (non-HR) clones than the HR clones (Maruyama et al., 2015).

### 3.4 Messenger RNA molecules expressing nls-I-SceI enzyme are useful for maintaining genome safety

When a plasmid expression vector is transfected to express the nls-I-SceI enzyme gene for intra-cellular cleavage, a DNA molecule of the plasmid vector can integrate at sites on host chromosomes, potentially causing disruption of the genomic structure in a patient receiving InCDC-mediated precision genome editing for therapy. Therefore, we attempted to use messenger RNA molecules expressing the nls-I-SceI enzyme gene.

**FIGURE 4:**
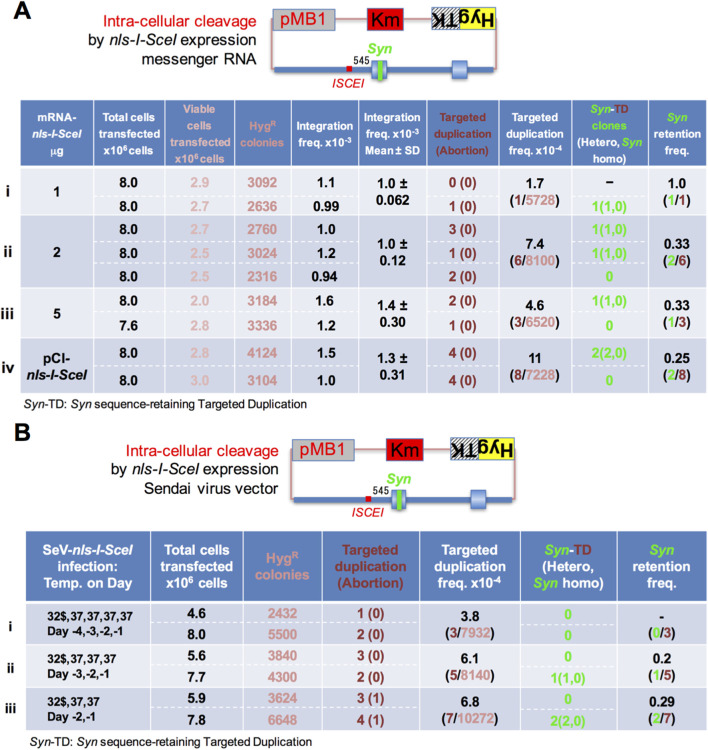
Messenger RNA molecules expressing intra-cellular cleaver nls-I-SceI lead to targeted duplication. **(A)** A circular donor plasmid, pMB1KmHygTK-HPRT(ISCEI(545)Ex2Syn), along with *nls-I-SceI*-expressing messenger RNA or the plasmid DNA expressing nls-I-SceI enzyme, shown in the first column, was transfected by electroporation into the number of total cells transfected, shown in the second column. In the next five columns, the numbers show the viable cells transfected, which were calculated by multiplying the number of total cells plated by the CFU (colony forming units)/500 total cells on two 9-cm-diameter dishes; the Hyg^R^ colonies generated; the overall integration frequency as the fraction of the Hyg^R^ colonies generated relative to the viable cells transfected; and the targeted duplication clones and the abortion-type clones in brackets, which are doubly positive and singly positive in the duplication test for Hyg^R^Tg^R^ colonies, respectively. In the last three columns, the frequency of the targeted duplication clones per overall integration (Hyg^R^) clone, the number of the *Syn*-retaining targeted duplication clones, and the frequency of the *Syn*-retaining targeted duplication clones per targeted duplication clone are shown. In these experiments, an advanced DMEM-based culture medium was used (*Materials and Methods*). The results from the allele sequence test are summarized in Supplementary Table 5. **(B)** A circular donor plasmid, pMB1KmHygTK-ISCEI(545)Syn, was transfected by electroporation into the HT1080 cells infected with the *nls-I-SceI*-expressing Sendai virus (SeV) vector, SeV-*nls-I-SceI*, which is prepared by infecting the SeV vector, amplifying at 32 °C for 1 day, and storing at −80 °C (*Materials and Methods*). Before electroporation, the stock of HT1080 cells with SeV-*nls-I-SceI* was thawed and incubated at 37 °C for indicated days, as shown in the first column. In the next three columns, the numbers show the total cells transfected, the Hyg^R^ colonies generated, and the targeted duplication clones and the abortion-type clones in brackets, which were doubly positive and singly positive in the duplication test for Hyg^R^Tg^R^ colonies, respectively. In the last three columns, the frequency of the targeted duplication clones per overall integration (Hyg^R^) clone, the number of the *Syn*-retaining targeted duplication clones, and the frequency of the *Syn*-retaining targeted duplication clones per targeted duplication clone are shown. In these experiments, a DMEM-based culture medium was used (*Materials and Methods*). The results from the allele sequence test are summarized in Supplementary Table 5.

When a circular donor plasmid pMB1KmHygTK-HPRT(ISCEI(545)Ex2Syn) was co-transfected into the recovered cells with 1, 2, or 5 μg of messenger RNA molecules expressing the *nls-I-SceI* gene, the targeted duplication clones were obtained at a frequency of 1.7–7.4 × 10^−4^ (1/5728 Hyg^R^, 6/8100 Hyg^R^, and 3/6520 Hyg^R^: lanes i, ii, and iii in Figure 4A) per overall integration clone, which was similar to that with the pCI-*nls-I-SceI* plasmid (8/7228: lane iv in Figure 4A). We confirmed that the frequencies of *Syn* retention per TD clone were 1/1, 2/6, and 1/3 (lanes i, ii, and iii in Figure 4A), which were similar to that with the pCI-*nls-I-SceI* plasmid (2/8: lane iv in Figure 4A).

We also attempted to use an RNA virus vector, a temperature-sensitive SeV vector (Ban et al., 2011), expressing the nls-I-SceI enzyme (Figure 4B). The HT1080 cells were infected with SeV18+nls-I-SceI/TS15ΔF, amplified, and stored at −80 °C (*Materials and Methods*). When a circular donor plasmid pMB1KmHygTK-HPRT(ISCEI(545)Ex2Syn) was transfected into the recovered cells with the SeV vector expressing nls-I-SceI cleaver, the targeted duplication clones were obtained at a frequency of 3.8–6.8 × 10^−4^ (3/7932 Hyg^R^, 5/8140 Hyg^R^, and 7/10272 Hyg^R^: lanes i, ii, and iii in Figure 4B) per overall integration clone, which were similar to those with messenger RNA molecules expressing the *nls-I-SceI* gene (lanes i, ii, and iii in Figure 4A), and the frequencies of *Syn* retention per TD clone were 0/3, 1/5, and 2/7 (lanes i, ii, and iii in Figure 4B), which were similar to those with messenger RNA molecules expressing the *nls-I-SceI* gene (lanes i, ii, and iii in Figure 4A).

These results indicate that messenger RNA or an SeV vector expressing the nls-I-SceI enzyme is useful for InCDC-mediated precision genome editing in clinical cells for cell therapy.

### 3.5 Demonstration for naturally generated replacement of the target sequence with the designed sequence

It is expected that a natural replacement clone (*HPRT* ^*+*^) will generate as a GCV^R^ colony among TD cells (*HPRT* ^*−*^; GCV^S^ background cells) incubated on a plated ware (Figure 1C); however, it is supposed that such desired GCV^R^ colonies will be decreased by intercellular bystander effects of GCV triphosphate molecules (Yang et al., 1998). The natural replacement clone should exhibit a HAT (hypoxanthine, aminopterin, and thymidine)-resistant phenotype (*HPRT* ^*+*^ = HAT^R^), whereas the TD background cells should exhibit a HAT-sensitive phenotype (*HPRT* ^*−*^ = HAT^S^). To verify whether GCV selects for natural replacement clones derived from targeted duplication cells, we first compared the frequency of GCV^R^ clones generated per viable cell with that of HAT^R^ clones to determine the accuracy of GCV selection for natural replacement (pop-out) clones, expressed as the ratio of GCV^R^/HAT^R^.

We used the HTG786 clone, a hetero-allelic (5′*SEXA1*/*Syn*3′) TD clone, which was obtained from the circular donor with HPRT(Ex2Syn(1195)ISCEI) (lane v in Figure 2A).

**FIGURE 5:**
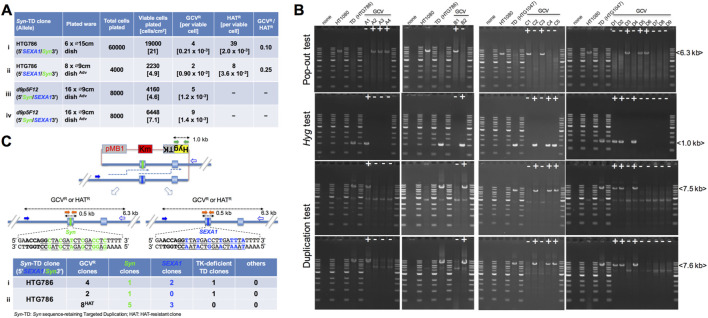
Demonstration for naturally generated replacement of the target sequence with the designed sequence. **(A)** *Syn*-retaining targeted duplication (*Syn*-TD) clone HTG786 (5′*SEXA1*/*Syn*3′) or a *Syn*-TD well clone *d9p5F12* (Figure 6A) is shown with a hetero-allele genotype in the first column. The total cells (third column) were plated onto the dishes (second column). The number of viable cells plated and the density of the viable cells (fourth column) are shown. GCV^R^/HAT^R^ (ganciclovir-resistant clones/hypoxanthine–aminopterin–thymidine-resistant (*HPRT* ^+^) clones; last column) is shown as the rate of the GCV^R^ frequency (fifth column) relative to that of the HAT^R^ frequency (sixth column). The viable cells plated were calculated by multiplying the number of total cells plated by CFU (colony forming units)/500 total cells. The density of viable cells was calculated by dividing the number of viable cells plated by the area (cm^2^) of the culture ware used. The GCV^R^ frequency was calculated by dividing the number of all GCV^R^ colonies generated by the viable cells plated. The HAT^R^ frequency was calculated by dividing the total number of HAT^R^ colonies generated or the total number of HAT^R^ colonies calculated from HAT^R^ colonies generated on two dishes by the number of viable cells plated. Adv: Advanced DMEM-based culture medium (*Materials and Methods*) was used to improve viability of HT1080-derived cells on culture ware. **(B)** Four GCV^R^ clones (GCV-A1–A4) derived from HTG786, two GCV^R^ clones (GCV-B1–B2) derived from HTG786, five GCV^R^ clones (GCV-C1–C5) derived from *d9p5F12* **(A)**, and nine GCV^R^ clones (GCV-D1–D9) derived from *d9p5F12* **(A)** were analyzed using three PCR-based assays as follows: (1) pop-out test (Figure 1E); (2) *Hyg* test (Figure 1E); and (3) duplication test (Figure 1D), with PCR products from each assay electrophoresed (the duplication test was performed to verify whether the duplication structure was retained). HTG786 is a TD clone with 5′*SEXA1*/*Syn*3’. HTG1047 is a TD clone with 5′*Syn*/*SEXA1*3’. **(C)** A TD structure with *SEXA1*/*Syn* hetero-allele sequences forms one of the two types of natural replacement structures: with the *Syn* allele (left) or with the *SEXA1* allele (right), which was verified by three PCR-based assays as follows: (1) pop-out test (closed blue arrow and open blue arrow) to verify the size characteristic of a natural replacement structure; (2) *Hyg* test (green arrows) to verify the absence of the *Hyg* gene because of the popping out of the circular plasmid DNA; and (3) allele sequence test (orange arrows) to determine the sequence of the allele of a replacement clone. One TD clone HTG786 is shown in the first column. In the following five columns, the numbers show the obtained GCV^R^ or HAT^R^ colonies, the *Syn* allele clones, the *SEXA1* allele clones, the TK-deficient TD clones, in which duplication tests were still positive in panel **(B)**, and the others (TD-derived deletion clones, in which all of pop-out test, *Hyg* test, and duplication test were negative in panel **(B)**). The complete results of the *Syn* allele or the *SEXA1* allele of pop-out-type GCV or HAT clones are summarized in Supplementary Table 6. The complete results of the *HygTK* sequence in TK-deficient TD-type GCV clones are summarized in Supplementary Table 7.

**FIGURE 6:**
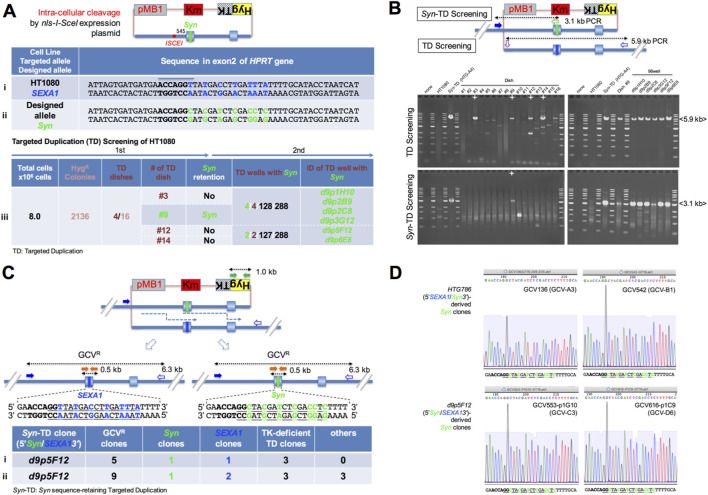
Verification of the generality of PCR screening methods to isolate the targeted duplication clones out of overall integration clones. **(A)** Upper panel: A circular donor plasmid, pMB1KmHygTK-HPRT(ISCEI(545)Ex2Syn) was transfected to convert the *SEXA1* allele of HT1080 to the *Syn* allele through intra-cellular circular donor cleavage (InCDC)-mediated targeted duplication (TD) and natural replacement. In line i, the sequence of the target allele (*SEXA1*) in the HT1080 cell line is shown, which has original codons (blue letters) in exon 2 of the *HPRT* gene. The bold upper-lined letters are the *SEXA1* site. In line ii, the sequence of the designed allele (*Syn*) is shown, which has synonymous codons (green letters) overlapped with the *SEXA1* site. Lower panel [Targeted duplication (TD) screening of HT1080]: In lane iii, in the first three columns, the numbers show the total cells transfected by electroporation and the hygromycin-resistant (Hyg^R^) colonies generated, and the TD dishes identified by PCR analysis for the bulk of the Hyg^R^ colonies on every dish of the 16 dishes are shown. In the next two columns, the ID of the TD dishes identified and the presence or absence of the *Syn* allele in each of the TD dishes are shown. In the last two columns, the numbers of *Syn*-TD wells/TD wells/cell-growing wells in three 96-well plates [upper: feeder (HT1080 cell line) wells; lower: feeder-free wells] and their ID of the *Syn*-retaining TD wells identified are shown. The viability of cells derived from a #9-dish stock in 96-well plates was not significantly improved by the coexistence with feeder cells (*Materials and Methods*). **(B)** Upper panel: The *Syn*-retaining TD dishes, which contain targeted duplication clones with *Syn* allele sequences, were screened using two PCR-based assays as follows: the TD-screening PCR with the plasmid-5′-inside forward primer (opened violet arrow) and the target-3′-outside reverse primer (opened blue arrow); the *Syn*-retaining TD-screening PCR with the target-5′-outside forward primer (closed blue arrow) and the *Syn* reverse primer (opened green arrow). Lower left panel: The circular donor plasmid, pMB1KmHygTK-HPRT(ISCEI(545)Ex2Syn), was transfected, and the resultant 16 dishes were screened by TD screening PCR and *Syn*-TD screening PCR, with both PCR products electrophoresed, leading to the identification of *Syn*-retaining TD dish #9. Lower right panel: Six wells of 96-well plates were identified as *Syn*-TD clones derived from #9-dish stock by TD screening PCR and *Syn*-TD screening PCR, with both PCR products electrophoresed, leading to the identification of six *Syn*-retaining TD wells. HTG-A4 is a TD clone with 5′*Syn*/*SEXA1*3’. **(C)** A TD structure with *Syn*/*SEXA1* hetero-allele sequences forms one of the two types of natural replacement structures: with the *Syn* allele (left) or with the *SEXA1* allele (right), which was verified using three PCR-based tests: (1) pop-out test (closed blue arrow and open blue arrow) to verify a natural replacement structure; (2) *Hyg* test (green arrows) to verify the popping out of the circular plasmid DNA; and (3) allele sequence test (orange arrows) to determine either allele of a replacement clone. One TD well-clone *d9p5F12* is shown in the first column. In the following five columns, the numbers show the obtained GCV^R^ colonies, *Syn* allele clones, *SEXA1* allele clones, and TK-deficient TD clones, in which duplication tests were still positive (Figure 5B), and others [TD-derived deletion clones, in which all of pop-out test, *Hyg* test, and duplication test were negative (Figure 5B)]. The complete results of the *Syn* allele or the *SEXA1* allele of pop-out type GCV clones are summarized in Supplementary Table 6. The complete results of the *HygTK* sequence of TK-deficient TD-type GCV clones are summarized in Supplementary Table 7. Upper panel: The sequence data from the *Syn* clones identified using the allele sequence test among HTG786-derived pop-out type GCV^R^ clones (lanes i and ii in Figure 5C) are shown, each of which was the singlet chart of the *Syn* allele sequence. Lower panel: The sequence data from the *Syn* clones identified using the allele sequence test among *d9p5F12*-derived pop-out-type GCV^R^ clones (lanes i and ii in Figure 6C) are shown, each of which was the singlet chart of the *Syn* allele sequence. The sequence data from their *SEXA1* clones are also shown in Supplementary Table 6.

When 1.9 × 10^4^ viable cells of HTG786 were plated at 21 cells/cm^2^ as a seeding viable cell density and selected by GCV medium or HAT medium from the fifth day after plating, four GCV^R^ colonies were observed; the generation of 39 HAT^R^ colonies was based on the data observed (lane i in Figure 5A). The frequency of each of the GCV^R^ clones and HAT^R^ clones per viable HTG786 cell plated is 0.21 × 10^−3^ and 2.0 × 10^−3^, respectively (GCV^R^/HAT^R^: 0.10). The resultant four GCV^R^ clones were analyzed using the pop-out test, *Hyg* test (Figure 1E), and duplication test (Figure 1D) so that three clones out of them were verified as the natural replacement (pop-out) type structure (GCV-A2, A3, and A4 in Figure 5B). To verify the generation of pop-out clones remaining the designed allele *Syn*, we analyzed these four GCV^R^ clones derived from HTG786 with the allele sequence test (Figure 1E). Out of four GCV^R^ clones, one clone had the *Syn* allele (upper of Figure 6D) and two clones had the *SEXA1* allele (lane i in Figure 5C). The remaining clone indicated the presence of the *Hyg* gene and the doublet configuration (GCV-A1 in Figure 5B), suggesting that it is a TK-deficient TD clone (lane i in Figure 5C). The accuracy of GCV selection for pop-out clones (0.075) is finally calculated by multiplying 0.1 (GCV^R^/HAT^R^) by 3/4 (pop-out/GCV^R^) when the seeding viable cell density is 21 cells/cm^2^.

Second, the cell suspension of HTG786 was plated at 4.9 cells/cm^2^ as a lower density of viable cells, considering intercellular bystander effects of GCV triphosphate molecules (Yang et al., 1998). GCV selection or HAT selection from day 5 after plating isolated two GCV^R^ colonies and eight HAT^R^ colonies (lane ii in Figure 5A). The frequency of each of the GCV^R^ clones and HAT^R^ clones per viable HTG786 cell plated is 0.90 × 10^−3^ and 3.6 × 10^−3^, respectively (GCV^R^/HAT^R^: 0.25). The resultant two GCV^R^ clones were analyzed using the pop-out test, *Hyg* test (Figure 1E), and duplication test (Figure 1D), and one of the clones was verified to have the natural replacement (pop-out) type structure (GCV-B1 in Figure 5B). To verify the generation of pop-out clones retaining the designed allele *Syn*, we analyzed the GCV^R^ clones using the allele sequence test (Figure 1E). Out of two GCV^R^ clones, one clone had the *Syn* allele (lane ii in Figure 5C; upper of Figure 6D). The remaining clone indicated the presence of the *Hyg* gene and the doublet configuration (GCV-B2 in Figure 5B), suggesting that it is a TK-deficient TD clone (lane ii in Figure 5C). We confirmed that the resultant eight HAT^R^ clones consisted of five *Syn* alleles and three *SEXA1* alleles and no TK-deficient TD clones were included (lane iii in Figure 5C). The accuracy of GCV selection for pop-out clones (0.13) is finally calculated by multiplying 0.25 (GCV^R^/HAT^R^) by 1/2 (pop-out/GCV^R^) when the seeding viable cell density is 4.9 cells/cm^2^.

These results indicate that several pop-out clones are possible to obtain through the GCV selection against more than 2,000 viable cells of a hetero-allelic TD clone and that the plating at densities as low as approximately 5 seeding viable cells/cm^2^ is useful to alleviate intercellular bystander effects of GCV triphosphate molecules (Yang et al., 1998).

### 3.6 Verification of the generality of PCR screening methods to isolate the targeted duplication clones out of overall integration clones

We suppose that human genome correction mediated by the InCDC technology of circular donor cleavage-mediated targeted duplication and natural replacement requires the following process:

1. Transfection of designed circular donor plasmid with an nls-I-SceI enzyme gene expression vector into human cells (TD1 and RI1 in Figure 1C)
2. Hygromycin selection of the targeted duplication clones (TD2 in Figure 1C) or the random integration clones (RI2 in Figure 1C)
3. PCR screening of TD clone-containing dishes for all of the dishes
4. PCR screening of TD clone-containing wells for all of the wells containing the cells derived from a TD clone-containing dish
5. Ganciclovir selection for natural replacement clones (TD3-1 and -2 in Figure 1C) derived from the TD clone (TD2 in Figure 1C)
6. Isolation of natural replacement (pop-out) clones with the designed sequence (TD3-1 in Figure 1C)

To verify that the above process leads to natural replacement clones with the designed sequence, we transfected with a circular donor plasmid, pMB1KmHygTK-HPRT(ISCEI(545)Ex2Syn) (*Syn* allele), and an nls-I-SceI expression plasmid into HT1080 cells (*SEXA1* allele) as the first step (lanes i and ii in Figure 6A) so that 2,136 Hyg^R^ colonies (overall-integration clones) were obtained on 16 dishes as the second step (lane iii in Figure 6A).

In the third step, to identify the TD dishes that contain targeted duplication clones are included, the Hyg^R^ colonies from each dish are created, and 16 frozen stocks are made. The genomic DNA is extracted from the cells derived from every dish and analyzed using PCR with the primer pair, plasmid-5′-inside forward primer and target-3′-outside reverse primer (TD screening in Figure 6B), to verify the structure of targeted duplication. Four TD-containing dishes (#3, 9, 12, and 14) out of 16 dishes were obtained (Figures 6A,B). To determine which of the four TD dishes contains *Syn* allele-retaining TD clones, the genomic DNA is also analyzed by PCR using the primer pair, target-5′-outside forward primer and *Syn* reverse primer (*Syn*-TD screening in Figure 6B). The #9 TD dish contained only *Syn*-TD clones (Figures 6A,B). Out of 16 dishes containing 2,136 overall integration clones as Hyg^R^ colonies, four dishes contained TD. Among these, only one dish contained *Syn*-TD. These results are in harmony with the frequencies of *Syn*-TD clones per overall integration clone [8/6,348 (0.1%): lane iii in Figure 2A; 4/8,448 (0.05%): lane i in Figure 3B; 2/7,228 (0.03%): lane iv in Figure 4A]. These frequencies are similar to those (0.02%) of HR clones per transfected cell obtained through CRISPR–Cas9 with oligonucleotide donor DNA in mammalian cells (Maruyama et al., 2015), implying that the screening sizes required to isolate desired HR clones are similar between InCDC and CRISPR–Cas9.

In the fourth step, to identify *Syn*-TD clones from TD dish stock #9 using 96-well plates, fresh cells derived from #9 TD dish stock were serially diluted, seeded at two cells per well into two sets of three 96-well plates, and then incubated in the presence of hygromycin. The resultant growing cells were observed in 128 and 127 wells, respectively (Figure 6A), and then analyzed by PCR using the primer pairs for TD screening and *Syn*-TD screening (Figure 6B) to verify *Syn*-retaining TD wells. Four wells (*Syn*-TD well *ID*: *d9p1H10*, *d9p2B9*, *d9p2C8*, and *d9p3G12*) out of 128 cell-growing wells and two wells (*Syn*-TD well ID: *d9p5F12* and *d9p6E8*) out of 127 cell-growing wells were identified as *Syn*-retaining TD clones (Figures 6A,B). In this study, we quantified the frequencies of InCDC-mediated donor-DNA overall integration events (mostly random integration events) per viable cell and obtained values of approximately 1 × 10^−3^ (Figures 3, 4). This represents the risk level that each screened *Syn*-retaining TD well clone contains a random integration fragment somewhere in the chromosomes.

In the fifth step, the cell suspension of a *Syn*-TD well clone *d9p5F12* was plated at 4.6 cells/cm^2^ as a low density of viable cells. GCV selection from the fifth day after plating isolated five GCV^R^ colonies (lane iii in Figure 5A). The frequency of GCV^R^ clones per viable *d9p5F12* cell plated is 1.2 × 10^−3^ (lane iii in Figure 5A). The resulting five GCV^R^ clones were analyzed using the pop-out test, *Hyg* test (Figure 1E), and duplication test (Figure 1D), and two of the clones were verified to have the natural replacement (pop-out)-type structure (GCV-C1 and C3 in Figure 5B).

To verify the generation of the pop-out clones retaining the designed allele *Syn*, we analyzed the GCV^R^ clones derived from *d9p5F12* for the allele sequence test (Figure 1E). Out of five GCV^R^ clones, one clone had the *Syn* allele (lower of Figure 6D) and one clone had the *SEXA1* allele (lane i in Figure 6C). The remaining three clones indicated the presence of the *Hyg* gene and the doublet configuration (GCV-C2, C4, and C5 in Figure 5B), suggesting that it is a TK-deficient TD clone (lane ii in Figure 6C). The other experiment with the same *Syn*-TD well clone *d9p5F12* (lane iv in Figure 5A and GCV-D1–D9 in Figure 5B) showed similar results [one clone: pop-out with the *Syn* allele (lower of Figure 6D); two clones: pop-out with the *SEXA1* allele; three clones: TK-deficient TD (lane ii in Figure 6C)] as the first experiment, except for the generation of three of TD-derived deletion clones (GCV-D7, D8, and D9 in Figure 5B and lane ii in Figure 6C).

A GCV^R^ colony-derived cell stock, which was obtained from each of these two GCV selection experiments against *d9p5F12*, contained clonally different cells consisting of a *Syn* clone and a TK-deficient TD clone or of a *Syn* clone and a *SEXA1* clone. Thus, additional 96-well cell cloning for the GCV^R^ cell stock (*Materials and Methods*) was needed, as described in Supplementary Tables 6, 7.

These results demonstrate that the positive selection and the two-step (dish-to-96-well) screening are successful in obtaining a single *Syn*-TD clone, and the subsequent negative selection with additional cell cloning leads to the isolation of a single desired clone through InCDC-primed precision genome editing.

## 4 Discussion

### 4.1 Double-strand break repair mechanisms induced by targeted genomic breakage

Targeted genomic breakage-related techniques have shown that the precise end-joining pathway, which is simple rejoining, was a rare event, and the microhomology-mediated end-joining pathway, in which the double-strand break has been filled with unexpected sequences or rejoined with a small deletion, was a frequent event in human cells (Maruyama et al., 2015). Targeted genomic breakage repeatedly occurs after the precise end-joining (Lin et al., 2013) so that the imprecise end-joining product is fixed to survive the host cell. That is the reason why these techniques mainly produce the microhomology-mediated end-joining products. A technique to regulate or attenuate CRISPR–Cas9 activity is useful to prevent repeated targeted breakage, leading to a decrease in imprecise end joining and genome toxicity (Kawamata et al., 2023). The current challenge of these techniques is to facilitate the desired pathway, such as the synthesis-dependent strand-annealing pathway, to achieve precise genome editing (genome correction).

### 4.2 Double-strand break repair mechanisms primed by InCDC

**FIGURE 7:**
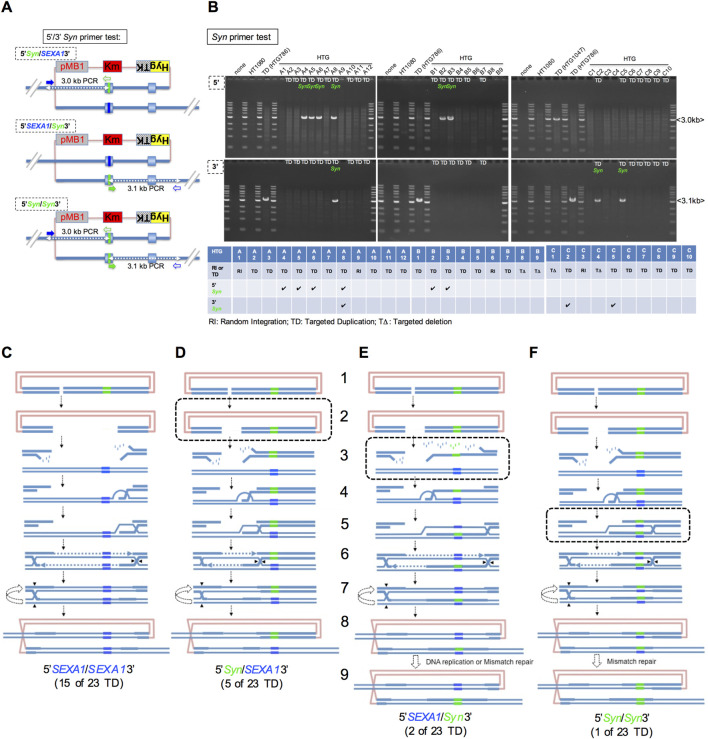
Structures of the Syn allele and the *SEXA1* allele in targeted duplication. **(A)** The *Syn* position of TD clones was verified by a couple of PCR-based tests as follows: (1) The 3.0-kb PCR with target-5′-outside forward primer 2 (closed blue arrow) and *Syn* reverse primer (opened green arrow: 5′-GCA​AAA​GAG​GTC​GAG​ATC​GTA​GCC-3′); (2) the 3.1-kb PCR with *Syn* forward primer (closed green arrow: 5′-GAA​CCA​GGC​TAC​GAT​CTC​GAC​CTC-3′) and target-3′-outside forward primer 2 (opened blue arrow). The 5′*Syn*/*SEXA1*3′ form leads to only the PCR product with the *Syn* reverse primer (upper). The 5′*SEXA1*/*Syn*3′ form leads to only the PCR product with the *Syn* forward primer (middle). The 5′*Syn*/*Syn*3′ form leads to both types of PCR products (bottom). **(B)** The PCR products obtained from the above two tests for HTG-A1–A12, HTG-B1–B9, and HTG-C1∼C10 (Figures 2B,C) are shown by electrophoresis, which shows that 15, 5, 2, and 1 out of 23 TD clones were 5′*SEXA1*/*SEXA1*3′, 5′*Syn*/*SEXA1*3′, 5′*SEXA1*/*Syn*3′, and 5′*Syn*/*Syn*3′, respectively. HTG786 is a TD clone with 5′*SEXA1*/*Syn*3’. HTG1047 is a TD clone with 5′*Syn*/*SEXA1*3’. **(C–F)** Crossover-type homologous recombination reactions produce four types of TD products: 5′*SEXA1*/*SEXA1*3′, 5′*Syn*/*SEXA1*3′, 5′*SEXA1*/*Syn*3′, and 5′*Syn*/*Syn*3’. Their reactions are explained as follows: 1. a donor plasmid DNA with a designed sequence (green) is cleaved at the *ISCEI* site within cells. 2. The double-strand break ends are resected by a putative exonuclease to form the double-strand gap. 3. The double-strand gap ends receive 5′-end resections to form the 3′-single-strand tails. 4. A 3′-single-strand tail is invaded into the homologous duplex of the chromosome to form the D-loop intermediate. 5. The D-loop intermediate is converted through strand exchange into the primed Holliday junction. 6. The repair synthesis starts at the 3′-end of the strand, which is stabilized in a structure of the primed Holliday junction to form the double Holliday junction. 7. The primed Holliday junction is horizontally resolved to form the single Holliday junction. 8. The single Holliday junction is vertically resolved to form the doublet structure comprising the donor DNA (green) and the chromosomal target DNA (blue), which possesses the donor allele duplex and the target allele duplex. Second on **(C,D):** A putative exonuclease degrades but remains the designed sequence of the donor DNA and forms the double-strand gap, which leads to the 5′*Syn*/*SEXA1*3′ doublet structure. However, when a putative exonuclease degrades beyond the designed sequence of the donor DNA and forms the longer double-strand gap, it leads to the 5′*SEXA1*/*SEXA1*3′ doublet structure. Third on **(E):** When the double-strand gap ends receive the 5′-end resections associated with longer processivity to form the longer 3′-single-strand tail, it leads to the doublet structure with the single mismatch (seventh and eighth on **(E)**) and produces the 5′*SEXA1*/*Syn*3′ doublet structure after a DNA replication fork passes or the mismatch is repaired (ninth on **(E)**). Fifth on **(F):** When the D-loop intermediate is converted through the branch migration associated with longer processivity to form another type of primed Holliday junction beyond the designed sequence, it leads to the doublet structure with the double mismatch (seventh and eighth on **(F)**) and produces the 5′*Syn*/*Syn*3′ doublet structure after the double mismatch is repaired (ninth on **(F)**).

The InCDC techniques have been programed to facilitate ends-in gene targeting crossover-type HR in host cells to produce the targeted duplication structures (Figures 1C,D). Examples of such a crossover-type HR-mediated targeted duplication had been reported as follows: (1) ends-in gene targeting into murine ES cells (Hasty et al., 1991) or human HT1080 cells (Brenneman et al., 1996) of linearized plasmid (*neo*) donor DNA; (2) ends-in gene targeting into *Drosophila melanogaster* of cleavable extrachromosomal donor DNA (Rong and Golic, 2000; Maggert et al., 2008); (3) ends-in gene targeting into *Saccharomyces cerevisiae* of linearized plasmid donor DNA (Hashida-Okado et al., 1998; Yamana et al., 2005; Miura et al., 2013). In this study, we verified the applicability to *ex vivo* precision genome editing (Figure 6) of InCDC-mediated crossover-type HR (targeted duplication) (Figures 7C–F). The current challenge of the InCDC techniques is to enhance a double-Holliday junction-mediated pathway for more efficient production of precision editing (genome correction). It is supposed that a putative exonuclease-attack occurs soon after a double-strand break and facilitates the formation of a broad-type double-Holliday junction, which stably connects the InCDC ends and its targeted locus (Figures 7C,D). Such a broad-type intermediate structure can protect against dissolution reactions of double Holliday structures, which produce non-crossover products (Wu and Hickson, 2003), thereby facilitating targeted duplication events. Thus, the safety distance from the *ISCEI* cleavage site to the designed sequence *Syn* protects the *Syn* sequence from a putative exonuclease attack and also serves to keep it outside a broad-type intermediate structure (third lane in Figure 7D), which facilitates *Syn*-retaining targeted duplication events (Figure 7D). The *HPRT* assay system, using the *Syn* allele and the *SEXA1* allele for InCDC-mediated human genome editing, helps identify enzymes involved in crossover-type homologous recombination in humans.

### 4.3 Naturally generated replacement of the target sequence with the designed sequence

A previous study (Brenneman et al., 1996) demonstrated meganuclease-induced pop-out events between the designed meganuclease-recognition sequence and the target DNA on the doublet configuration, which was intervened by plasmid DNA containing a positive selection gene (*neo*) in human cells. Other previous studies (Rong and Golic, 2000; Maggert et al., 2008) demonstrated the break-induced replacement events between the designed donor DNA and the target DNA in *Drosophila*. In mice, the natural replacement events of a duplication structure had been verified (Wu et al., 1994). In human HT1080 cells, we demonstrated that natural replacement clones could be isolated by negative selection without any artificial breakage in the doublet configuration, which was intervened by plasmid DNA bearing a positive–negative selectable fusion gene (*HygTK*). This natural property is supposed to be related to the popping out of the circular DNA, consisting of the plasmid DNA and either donor or target DNA, via DNA replication slippage (Viguera et al., 2001). In this study, we quantified the frequency of popping-out events per viable cell of a TD clone and obtained the values of more or less 3 × 10^−3^ in the 5.4-kb duplicative DNA regions (Figure 1E) based on the frequencies of HAT^R^ clones generated per viable TD cell (lanes i and ii in Figure 5A), which may indicate frequencies of DNA replication slippage.

## 5 Summary

A set of intra-cellular circular donor DNA and its cleaver enabled precision genome editing in human cells through TD, followed by natural replacement of the target sequence with the designed sequence. The safety distance from the cleavage site recognized by its intra-cellular cleaver to the designed sequence is crucial to protect the designed sequence from enzymatic exclusion. The two-step (dish-to-96-well) screening for overall-integration clones selected positively is successful in obtaining a single designed sequence-retaining TD clone. The screening size of each of the two steps is 16 dishes and several 96-well plates, respectively. The efficient natural replacement requires GCV selection on a low density of several thousand viable cells of a designed sequence-retaining TD clone. Thus, we suggest that the InCDC technology enables *ex vivo* precision genome editing in clinical cells at feasible screening sizes, followed by cell therapy.

## Data Availability

The datasets supporting the findings in this study are available in Zenodo at https://zenodo.org/records/18861092.
